# Comparison of Pulmonary Nodule Morphology on Pre-Contrast and Delayed Post-Contrast Stack-of-Spirals Ultrashort Echo Time MRI

**DOI:** 10.3390/diagnostics16193170

**Published:** 2026-09-29

**Authors:** Qing Fu, Peng-Wei Shu, Ren-Qiang Yu, Jia-Wei Wu, Zi-Kai Li, Ting Yin, Thomas Benkert, Jing Peng, Hao-Hao Lu, Fa-Jin Lv

**Affiliations:** 1 Department of Radiology, The First Affiliated Hospital of Chongqing Medical University, Chongqing 400016, China; fuqing2019@hust.edu.cn (Q.F.);; 2 Department of Radiology, Union Hospital, Tongji Medical College, Huazhong University of Science and Technology, Wuhan 430022, China; spw@hust.edu.cn (P.-W.S.);; 3 General Hospital of the Yangtze River Shipping, Wuhan Brain Hospital, Wuhan 430022, China; 4 MR Collaborations, Siemens Healthineers Ltd., Chengdu 610000, China; 5 MR Application Predevelopment, Siemens Healthcare Ltd., 91052 Erlangen, Germany; 6 Clinical & Technical Support, Philips Healthcare, Beijing 100600, China

**Keywords:** MRI, ultrashort echo time, pulmonary nodules, CT, morphology

## Abstract

**Background**: Stack-of-spirals ultrashort TE (spiral-UTE) MRI has shown promise for radiation-free pulmonary nodule detection, and previous studies have reported closely matched observed pulmonary nodule detection rates between contrast-enhanced (UTEe) and unenhanced (UTEu) acquisitions. However, whether this performance extends to detailed lesion-level morphological characterization and whether delayed post-contrast spiral-UTE provides additional morphological information compared with the pre-contrast acquisition under the clinical imaging protocol remain incompletely understood. Therefore, this study compared UTEu and UTEe for lesion-level morphological assessment using CT as the reference standard. **Methods**: A total of 61 patients with pulmonary nodules/masses (>5 mm) identified on chest CT were enrolled. Patients underwent spiral-UTE MRI on either a 1.5 T scanner (27 patients, 63 lesions) or a 3.0-T scanner (34 patients, 101 lesions). Two fixed consensus reader panels with different experience profiles evaluated lesion visualization and detailed morphological features separately on sequential pre-contrast UTEu and delayed post-contrast UTEe. Two complementary morphology analyses were performed. First, end-to-end successful characterization was assessed across all CT-detected eligible lesions; for each feature, successful characterization required both lesion visualization on the corresponding UTE acquisition and correct feature classification relative to CT. Nonvisualized lesions were designated not characterizable and therefore unsuccessful for this composite outcome. Second, conditional sensitivity, specificity, and accuracy were evaluated among lesions visualized on both UTEu and UTEe to compare morphology interpretation after successful lesion visualization. Lesion size agreement, contrast-to-noise ratio (CNR), and edge sharpness were also evaluated. **Results**: CT identified 164 lesions, of which the experienced consensus panel visualized 113/164 lesions (68.9%) on both UTEu and UTEe, three on UTEu only, three on UTEe only, and 45 on neither; the inexperienced panel visualized 93/164 lesions (56.7%) on UTEu and 91/164 (55.5%) on UTEe; 91 lesions were visible on both acquisitions, two on UTEu only, none on UTEe only, and 71 on neither. In field-strength-stratified end-to-end analyses, successful-characterization rates ranged from 55 to 71% for UTEe, and 51 to 71% for UTEu derived from experienced reader panel. Among common-visible lesions, no statistically detectable UTEu–UTEe differences were demonstrated for most evaluated morphological features. **Conclusions**: Across all CT-detected lesions, the ability of spiral-UTE to accurately characterize pulmonary nodule morphology was constrained by incomplete lesion visualization. Our analyses did not demonstrate a statistically detectable UTEu–UTEe difference for most evaluated morphological features. Among UTE-visible lesions, both acquisitions showed relatively high accuracy for several morphological features.

## 1. Introduction

Lung cancer remains a leading global health threat, with its morbidity and mortality ranking first among all malignancies worldwide [1]. The 2022 Global Cancer Statistics (updated by the International Agency for Research on Cancer) [2] noted it as the most commonly diagnosed cancer in 2022 (nearly 2.5 million new cases, 12.4% of all cancers) and the top cancer-related deaths cause (~1.8 million fatalities, 18.7% of total). Advanced lung cancer has a poor prognosis (5-year survival rate of <10%) [3], underscoring the critical role of early detection, accurate diagnosis and precise treatment in improving patient outcomes [4].

Chest computed tomography (CT) dominates clinical imaging for pulmonary nodules/masses. Its ability to identify morphologic features (e.g., spiculated margins, lobulated borders, increased size, and large solid components) enables noninvasive risk stratification of malignancy potential across solid, subsolid, and pure ground-glass nodules [5,6,7,8,9]. While low-dose chest CT reduces radiation exposure, cumulative radiation risks persist—particularly in vulnerable populations (pregnant women, youth, children, and patients requiring repeated follow-ups) [10,11]. By contrast, ultrashort echo time (UTE) MRI has emerged as a promising radiation-free technique for lung assessment; ultrashort echo time (UTE) magnetic resonance imaging (MRI) effectively captures rapidly decaying lung signals and minimizes susceptibility and motion artifacts, complementing CT in pulmonary nodule detection and classification, with comparable diagnostic performance [12,13,14,15]. Wielpütz et al. [16] evaluated respiratory-gated radial UTE (7 min acquisition) against CT in 119 lesions, reporting 92.4% sensitivity for specific morphological features and moderate-to-substantial intermodality agreement, validating UTE’s role in nodule characterization.

Recent advances have led to the development of free-breathing stack-of-spirals UTE (spiral-UTE), which combines Cartesian slice selection and spiral in-plane encoding for efficient k-space coverage [17,18]. Compared with radial acquisition, spiral-UTE offers superior motion robustness, higher readout efficiency [19,20], and preserved image quality [19,21,22,23,24], especially in patients unable to perform adequate breath-holding. In contrast, the relatively inefficient k-space sampling of radial acquisition often leads to prolonged scan time, which exacerbates motion-related artifacts and compromises the acquisition of high-quality clinical lung images [25,26]. Its clinical utility is supported by the following high detection rates: 90.8% for pulmonary metastases in breast cancer patients [24] and 82.61% for nodules <6 mm [27]. Integrating iterative self-consistent parallel imaging reconstruction (SPIRiT) further enhances reconstruction performance and shortens scan time [28], addressing a key barrier to clinical adoption.

Existing spiral-UTE studies for pulmonary nodule detection (vs. CT) have used either contrast-enhanced (UTEe, gadolinium-enhanced) [20,24,29] or unenhanced (UTEu) protocols [19,21,27,29] across 1.5 T and 3.0 T scanners. As paramagnetic agents, gadolinium-based contrast agents enhance the signal intensity of vascularized tissues on MRI [29]. Following intravenous administration, they diffuse into the microvasculature of lesions and exhibit variable retention based on vascular permeability and blood–tissue barrier integrity, helping to clearly delineate lesion boundaries, internal vascular supply characteristics, and the extent of adjacent tissue invasion [30,31], allowing early and accurate diagnosis, appropriate treatment planning, as well as the monitoring of disease progression. Previous spiral-UTE studies have primarily focused on pulmonary nodule detection and overall lung image quality using either UTEu or UTEe acquisitions. Our previous study [29] has directly compared the image quality of UTEu and UTEe at 1.5 T and evaluated their ability to detect pulmonary nodules relative to CT. However, less is known about their performance for the subsequent morphological characterization of CT-detected nodules, for which both lesion visualization and accurate feature assessment are required. In addition, whether delayed post-contrast UTEe improves this overall characterization performance compared with pre-contrast UTEu also remains unclear.

Therefore, the principal objective of this study was to evaluate the ability of pre-contrast UTEu and delayed post-contrast UTEe to visualize and characterize pulmonary nodules using CT as the reference standard across all eligible CT-detected lesions. To distinguish overall clinically achievable performance from morphology interpretation after successful visualization, our secondary objectives were to compare morphological characterization between UTEu and UTEe among UTE-visible lesions and to evaluate lesion-size measurement, quantitative image characteristics, reader-panel findings, and field-strength cohorts.

## 2. Materials and Methods

### 2.1. Study Population

This study was approved by the institutional ethics committee and conducted in accordance with the Declaration of Helsinki. The written informed consent was obtained from all the patients.

Between May 2024 and August 2026, patients with CT-detected suspected pulmonary nodules/masses were recruited. These patients were already scheduled for gadolinium-enhanced head/abdominal MRI to evaluate metastasis and prospectively underwent additional unenhanced (UTEu) and contrast-enhanced (UTEe) spiral-UTE scans for study inclusion. The time delay between contrast injection and UTEe acquisition was 10–15 min, which was not standardized, potentially introducing bias. The inclusion criteria were: (a) presence of single/multiple pulmonary nodules (>5 mm) on chest CT, nodules < 5 mm were excluded, because the primary objective of this study was detailed morphological characterization rather than small-nodule detection, and reliable assessment of multiple morphological features is inherently limited for very small lesions at the spatial resolution of the current spiral-UTE acquisition. While UTE’s performance in detecting small nodules remains variable [prior rates: 11–17% [32,33] vs. 74.1% for 4–6 mm and 76.7–88.1% for 2–5 mm [13,20,24]], this exclusion was justified by our primary objective of comparing UTEu and UTEe for morphological assessment; (b) time interval between clinically required contrast-enhanced head/abdominal MRI and chest CT ≤ 4 days; and (c) willingness to undergo additional free-breathing UTEu (pre-gadolinium) and UTEe (post head/abdominal MRI). The exclusion criteria were: (a) contraindications to gadolinium-based contrast-enhanced MRI; (b) refusal to undergo additional spiral-UTE scans; (c) two experienced radiologists jointly evaluated the chest CT/spiral-UTE images—the case would be excluded from the current study if the images were inadequate to reach diagnostic purpose; and (d) presence of pulmonary calcified nodules, because MRI is less effective for detecting pulmonary calcified nodules because both calcifications and lung parenchyma exhibit low signal intensity on MRI. This cohort was prospectively recruited independently of that included in our previously published study [29] of spiral-UTE lung imaging. There was no overlap in enrolled patients, CT or MRI examinations, source imaging datasets, lesion-level data, or prospectively collected study data between the two studies.

### 2.2. Lung MRI Protocol

All the examinations were performed on two MR scanners, including one 1.5 T (Magnetom Aera, Siemens Healthcare, Erlangen, Germany) equipped with a 45 mT/m achievable gradient strength and 200 T/m/s maximum slew rate and one 3.0 T (Magnetom Skyra, Siemens Healthcare, Germany) equipped with a 45 mT/m achievable gradient strength and 200 T/m/s maximum slew rate; a dedicated 18-channel body coil combined with a 32-channel spine coil was used for lung coverage. Participants were instructed to breathe regularly (no breath-hold) before scans. UTEu was acquired first in coronal orientation before contrast administration. Intravenous gadobutrol (Gadovist; Bayer Schering Pharma, Berlin, Germany) was subsequently administered at 0.1 mL/kg body weight at an injection rate of 1.5 mL/s, followed by a saline flush. The clinically indicated contrast-enhanced head or abdominal MRI sequences were then performed, after which UTEe was acquired in the same coronal orientation. UTEe was obtained approximately 10–15 min after contrast administration; this post-injection interval was not strictly standardized because the intervening clinical examinations differed among patients.

UTEu and UTEe used identical spiral-UTE acquisition parameters as follows: TR (repetition time): 3.26 ms, TE (echo time): 0.05 ms, FOV (field of view): 512 × 512 mm^2^, slice thickness: 1.6 mm, number of slices: 120, matrix size: 320 × 288, flip angle: 5°, voxel size: 1.6 × 1.6 × 1.6 mm^3^, acquisition orientation: coronal, spiral-out duration: 1160 μs, acceleration factor in parallel imaging: 2, reconstruction mode: iterative self-consistent parallel imaging reconstruction from arbitrary k-space (SPIRiT), TA (acquisition time): averaged 4 min depending on patients’ breathing pattern, and respiratory mode: free-breathing (no breath-hold).

However, because UTEu was always acquired before UTEe, acquisition order was fixed rather than randomized or counterbalanced. Therefore, the comparison between UTEu and UTEe represents a paired comparison between a pre-contrast acquisition and a delayed post-contrast acquisition obtained later during the same examination and does not independently isolate the effect of gadolinium administration from potential effects of elapsed examination time, respiratory variability, motion, fatigue, or delayed enhancement kinetics.

### 2.3. Chest CT Protocol

Clinically indicated unenhanced chest CT was served as the reference standard, with ≤4 days interval from spiral-UTE. CT examinations were performed using a standardized chest CT protocol. Chest CT scans were conducted on three CT scanners (Definition AS+ and Force, Siemens Healthcare, Germany) with patients in breath-hold and arms raised. The CT parameters included a 120 kV tube voltage, a modulated tube current, 150–250 mA, a patient-adapted field of view, a pitch of 1.2, and 1 mm for transverse slice thickness reconstructions via B31f (mediastinal window) and B70f (lung window) kernels. Scans were performed at end-inspiration. The mean dose-length product for chest CT was 220.5 ± 31.1 mGy·cm and the mean effective dose was 3.1 ± 0.4 mSv.

### 2.4. Image Analysis

Two senior radiologists (>20 years of experience) independently reviewed chest CT images to reach consensus. They identified pulmonary nodules > 5 mm and characterized their morphological features using mediastinal (width: 400 HU; level: 20 HU) and lung (width: 1500 HU; level: −700 HU) windows. Discrepancies were resolved via discussion, with the final consensus serving as the reference standard. Spiral-UTE images were evaluated by two independent groups. Group 1 (experienced): two radiologists (10–15 years of experience) jointly reviewed UTEu and UTEe images, documenting visible nodules > 5 mm, their maximum diameter, and morphology. Disagreements were resolved via discussion. Group 2 (inexperienced): two resident radiologists (1–3 years of experience) conducted identical consensus-based reviews, documenting nodules > 5 mm and their morphology. Disagreements were also resolved via discussion. Representative disagreements involved subtle lesion conspicuity, differentiation of GGOs from adjacent vessels, classification of pure versus mGGOs, and delineation of internal low-signal components in lesions with heterogeneous signal intensity. Because the final analyses were based on consensus assessments within each reader pair, comparisons between the experienced and inexperienced panels represent comparisons between two fixed consensus panels rather than independent estimates of the effect of reader experience.

For the measurement of the maximum diameter of each lesion, the largest diameter value was measured on the axial image at the slice showing the maximum lesion dimension. The measurement was repeated three times, and the averaged value was used for subsequent data analysis. No communication occurred between the two consensus panels during image interpretation. Each group underwent assessment with one spiral-UTE sequence (UTEu or UTEe) at each time point, with the second sequence evaluated 4 weeks later in randomized order to reduce memory bias. All evaluations were performed on a commercial CT/MR post-processing workstation; multiplanar reconstruction (MPR), curved planar reconstruction (CPR), and/or maximum intensity projection (MIP) were generated if needed.

### 2.5. Morphological Feature Assessment

Referring to previous reports [5,7,16], nodular morphological features were assessed separately on CT, UTEu, and UTEe images. Evaluated features included: nodular type (solid, ground glass opacity [GGO] or mixed GGO [mGGO]), margin (smooth, lobulation, spiculation and/or irregular), dominant shape (round or oval), cavitation (central hypodense/hypointensity of lesion, perilesional lucency and air-bronchogram), relationship with surrounding vessels (vessels passing through, passing by, distorted or dilated vessels), and pleural interrelationship (interlobular fissure invasion, pleural adhesion, pleural retraction or pleural effusion).

To quantify two complementary aspects of spiral-UTE morphology performance, two analyses were performed. First, an end-to-end successful-characterization outcome was evaluated across all CT-detected eligible lesions. For each morphological feature and UTE acquisition, a lesion was considered successfully characterized when it was visualized on that acquisition and the corresponding feature classification agreed with CT. A nonvisualized lesion was designated not characterizable and therefore did not count as a successful characterization; however, it was not assigned an arbitrary feature-specific false-positive or false-negative classification.

Our secondary exploratory analysis evaluated conditional morphology-level performance among lesions visualized on both UTEu and UTEe. This common-visible analysis allowed direct paired comparison of morphology interpretation between UTEu and UTEe after successful lesion visualization.

### 2.6. Quantitative Analysis (CNR and Edge Sharpness)

Lesion-specific CNR and edge sharpness were evaluated as quantitative measures of lesion conspicuity and boundary delineation, respectively, for comparison between the sequential pre-contrast and delayed post-contrast acquisitions.

In the present study, lesion contrast refers to the signal-intensity difference between the pulmonary lesion and the low-signal airway reference, whereas CNR normalizes this signal difference by the empirical local signal variability represented by SD_airway_.

CNR [25] was calculated using the formula:

$$\mathrm{CNR}\;=\;\frac{{SI}_{lesion}-{SI}_{airway}}{{SD}_{airway}}$$

where SI_lesion_ referred to the average signal intensity (SI) of pulmonary nodules, measured via a region of interest (ROI) placed within the nodule while avoiding margins, internal hypodensity/hypointensity, or vessels. SI_airway_ was calculated as the mean SI values of three ROIs placed separately in the trachea, left main bronchus, and right main bronchus; ROIs were placed at identical locations for UTEu and UTEe. The airway ROIs were selected as low-signal reference regions within the thoracic field of view and were placed at corresponding anatomical locations on UTEu and UTEe images. SD_airway_ was defined as the mean of the within-ROI signal-intensity SDs obtained from the trachea and bilateral main bronchi and was used as an empirical surrogate of local image variability rather than a rigorous estimate of true image noise.

Because parallel imaging and iterative SPIRiT reconstruction may introduce spatially varying noise characteristics, the noise level was not estimated from a single ROI. Instead, the SD was measured separately in the trachea, left main bronchus, and right main bronchus, and the mean of these three measurements was used for CNR calculation. This approach was intended to reduce the influence of local spatial variations in image noise.

Edge sharpness was measured using methods adapted from previous reports [34,35]. A 1-pixel-wide line was placed across the nodule to the adjacent lung parenchyma boundary to generate a plot profile. ImageJ software 1.53t (http://imagej.nih.gov/ij/index.html, accessed on 24 March 2026) was used to record the maximum (Max) and minimum (Min) SI values from the profile. The distance (Dis) between two points on the profile, [0.8 × (Max − Min) + Min] and [0.2 × (Max − Min) + Min], was calculated. Edge sharpness was evaluated as a secondary exploratory quantitative measure of lesion-boundary delineation. Measurements were manually performed by one radiologist with 8 years of experience. For each lesion and acquisition, the line-profile measurement was repeated 2–3 times, and the mean value was used for subsequent analysis to reduce within-procedure variability related to manual measurement. These repeated measurements were not intended as a formal intraobserver reproducibility assessment.

### 2.7. Statistical Analysis

All statistical analyses were performed using IBM SPSS 21.0 statistical software. The distribution of continuous variables was assessed using the Shapiro–Wilk test together with visual inspection of Q–Q plots. Approximately normally distributed continuous variables are presented as mean ± standard deviation (SD), whereas non-normally distributed variables are presented as median and interquartile range (IQR). Categorical variables are presented as frequencies and percentages. For comparisons of patient-level characteristics between the 1.5- and 3.0-T cohorts, the two-sample *t* test was used for approximately normally distributed continuous variables, and the Mann–Whitney U test was used for non-normally distributed continuous variables. Categorical variables were compared using the chi-square test or Fisher’s exact test. For lesion-level comparison according to field-strength cohort, mixed-effect models were used with patient-specific random intercepts; linear, logistic, or multinomial mixed-effect models were used according to outcome type.

The primary analysis evaluated pulmonary nodule characterization across all CT-detected eligible lesions. For each morphological feature, end-to-end successful-characterization rates were defined as both visualization of the lesion and correct feature classification relative to the number of CT-detected eligible lesions. Visualization coverage was calculated as the proportion of CT-detected lesions identified on each UTE acquisition. Both rates were calculated separately for UTEu and UTEe within each reader panel and field-strength cohort. In the secondary analysis, sensitivity, specificity, and accuracy for morphological features were calculated among lesions visualized on both UTEu and UTEe by experienced reader panel. Because multiple lesions could arise from the same patient, 95% confidence intervals (95% CIs) were estimated using patient-level cluster bootstrap resampling.

For lesion-size analysis, maximum diameters measured on CT, UTEu, and UTEe were compared using the Friedman test with multiplicity-adjusted pairwise comparisons. Agreement with CT was assessed using Bland–Altman analysis. Absolute measurement errors relative to CT were also calculated for UTEu and UTEe and compared using the same patient-cluster bootstrap procedure. Linear regression was used to explore the association between CT lesion size and CT–UTE measurement discrepancy. Agreement between the two consensus reader panels was assessed using kappa statistics. A two-sided *p* value < 0.05 was considered statistically significant.

## 3. Results

### 3.1. Patient and Lesion Characteristics

**Figure 1:**
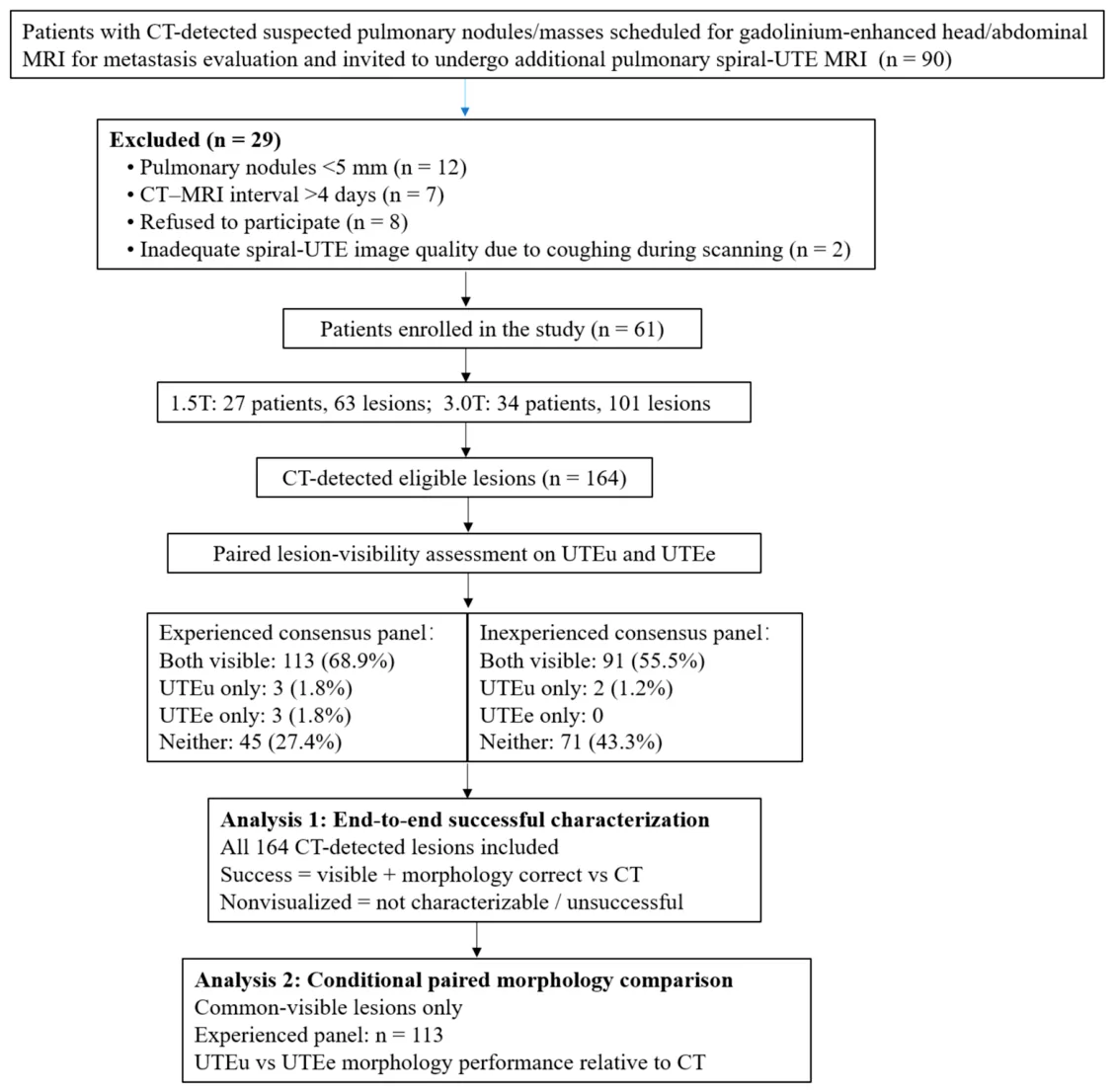
Flowchart of patient selection and the two-level lesion-analysis framework.

A total of 61 patients (35 males, 26 females; mean age, 58.4 ± 10.4 years; and age range, 19–74 years, Table 1) were enrolled (Figure 1). Twenty-seven patients with 63 lesions were examined at 1.5 T, whereas 34 patients with 101 lesions were examined at 3.0 T. Among the 164 CT-detected lesions, 113 were identified on both UTEu and UTEe by experienced consensus panel. These lesions constituted the population available for the experienced-panel conditional morphological comparison. The inexperienced consensus panel visualized 93 lesions on UTEu and 91 lesions on UTEe. Among the 45 CT-detected lesions not visualized by the experienced consensus panel, 14 were mixed GGO, 18 were pure GGO, and 13 were solid lesions; 27 were adjacent to the diaphragm, 14 to the heart, and four to the pleura.

### 3.2. End-to-End Characterization and Visualization Coverage

Among all 164 CT-detected eligible lesions, the experienced consensus panel visualized 116 lesions (70.7%) on UTEu and 116 (70.7%) on UTEe. Lesion-level pairing showed complete concordance between the two acquisitions as follows: 113 lesions (68.9%) were visible on both UTEu and UTEe, three (1.8%) on UTEu only, three (1.8%) on UTEe only, and 45 lesions (27.4%) were visible on neither acquisition (Figure 1). The inexperienced panel visualized 93/164 lesions (56.7%) on UTEu and 91/164 (55.5%) on UTEe; 91 lesions (55.5%) were visible on both acquisitions, two (1.2%) on UTEu only, none on UTEe only, and 71 (43.3%) on neither (Figure 1).

**Figure 2:**
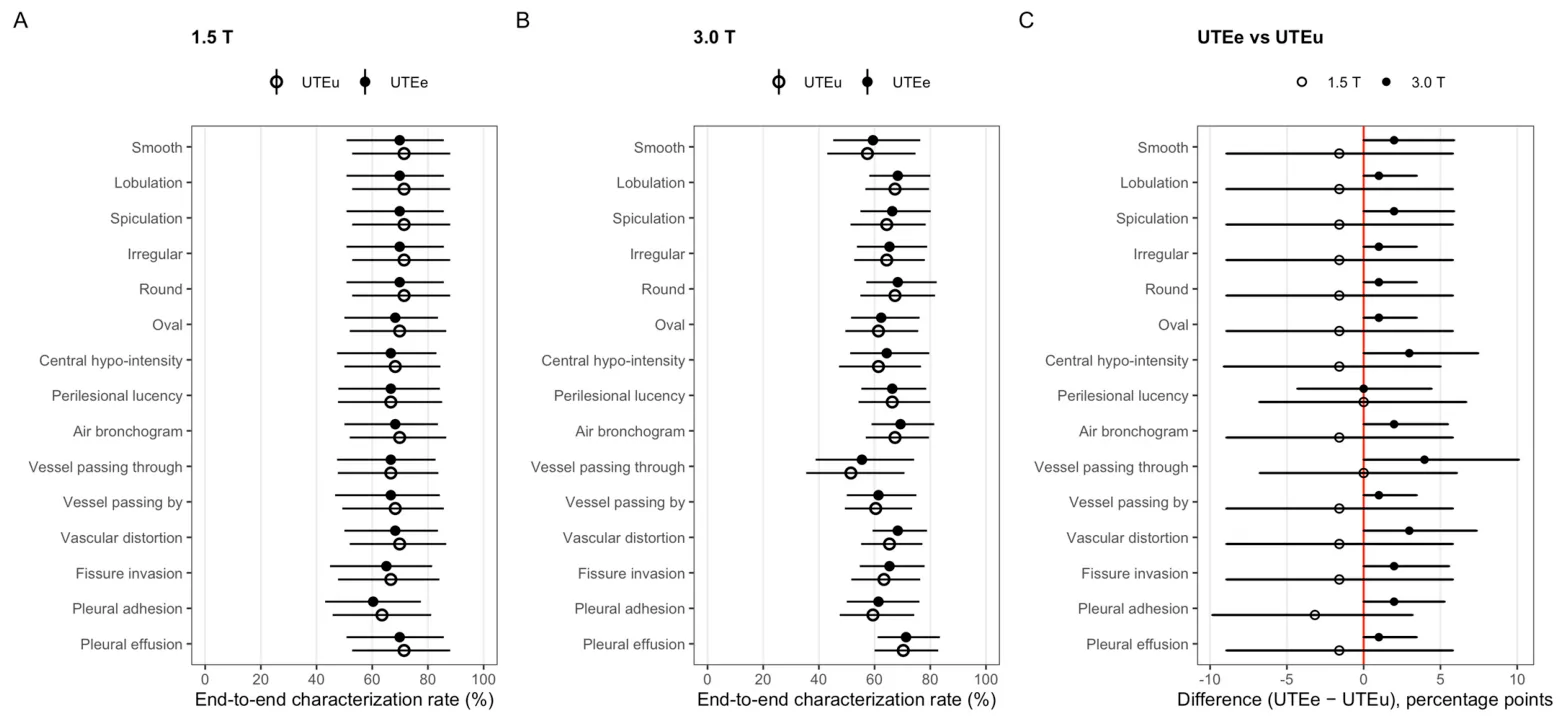
The end-to-end characterization rates of pulmonary nodule morphological features between UTEu and UTEe. Points represent estimated successful-characterization rates and horizontal bars represent 95% confidence intervals obtained using patient-level cluster bootstrap resampling. (**A**) 1.5 T cohort; (**B**) 3.0 T cohort; (**C**) paired absolute differences between UTEe and UTEu (UTEe − UTEu), with the red vertical reference line indicating the null difference (0 percentage points) between UTEe and UTEu.

**Figure 3:**
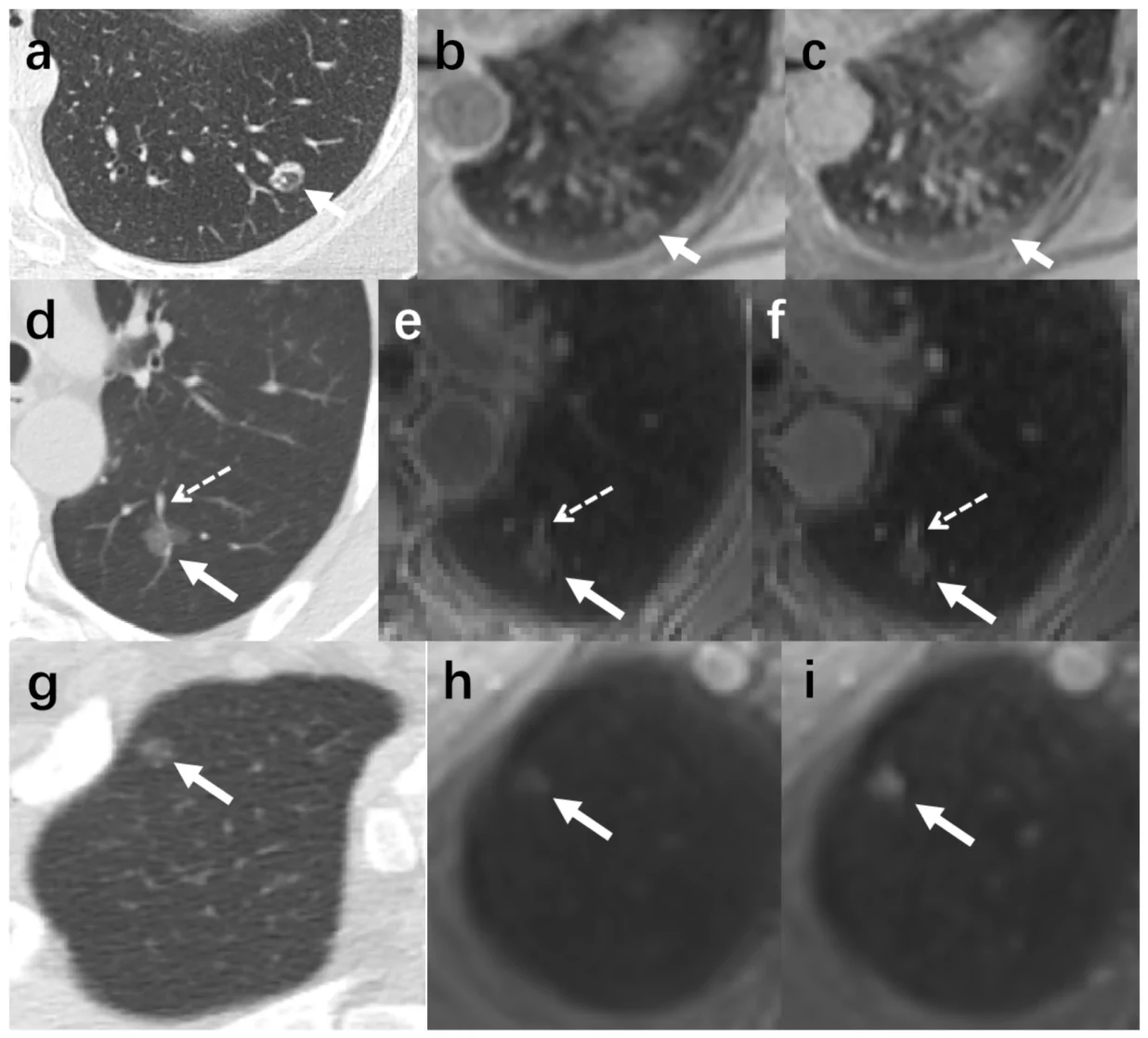
A patient with confirmed breast cancer with multiple pulmonary nodules. A part-solid nodule measuring 11.5 mm in diameter in left lower lobe was identified on chest CT (**a**), UTEu (**b**) and UTEe (**c**) with inhomogeneous signal intensity. A pure ground-glass opacity (GGO) in the left lower lobe measuring 10.0 mm was detected on chest CT (**d**), UTEu (**e**) and UTEe (**f**) (indicated by white solid arrows), which was correctly identified by the experienced consensus panel but was misinterpreted as a pulmonary vessel bifurcation by the inexperienced consensus panel (indicated by white dotted arrows). Another pure GGO in the right upper lobe measuring 5.4 mm was also clearly shown on chest CT (**g**), UTEu (**h**) and UTEe (**i**), but was defined as pure GGO on (**h**) and mixed ground-glass opacity nodule on (**i**) by the inexperienced consensus panel.

**Figure 4:**
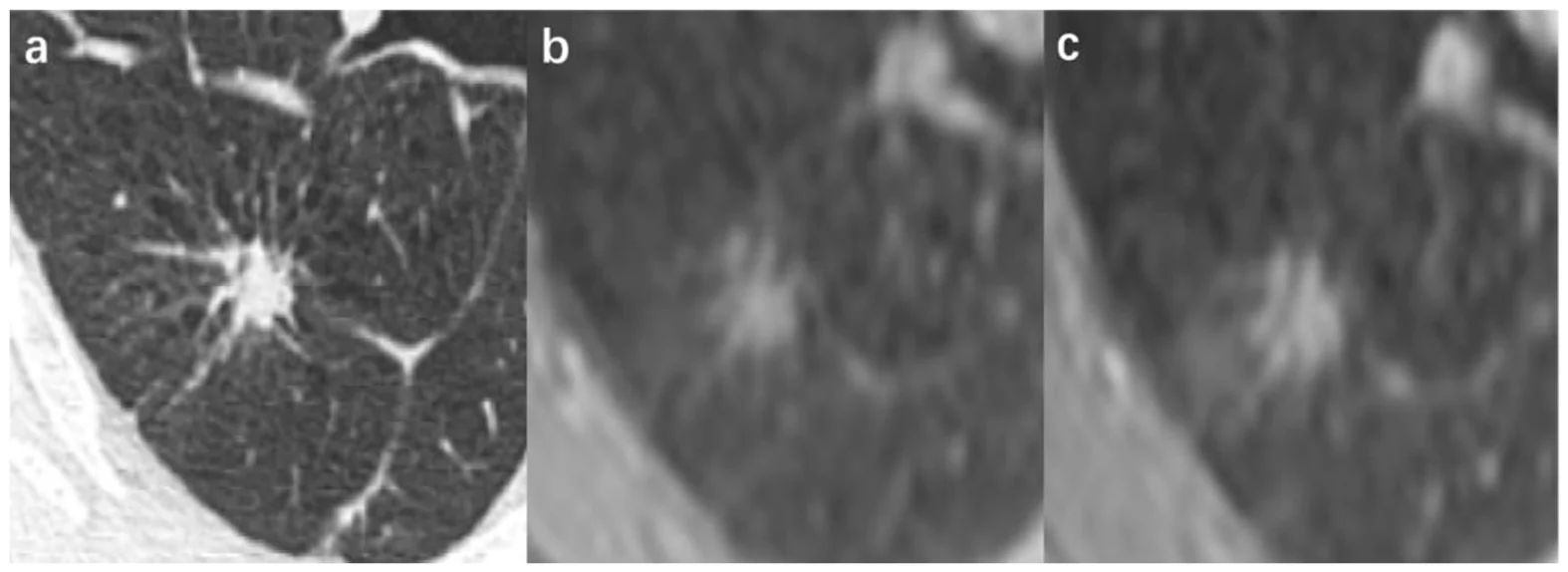
CT (**a**), unenhanced (**b**) and enhanced (**c**) spiral-UTE images obtained in a patient with identified peripheral lung cancer. Spiral-UTE images demonstrate an irregular shape and spiculated margins, consistent with CT finding.

**Figure 5:**
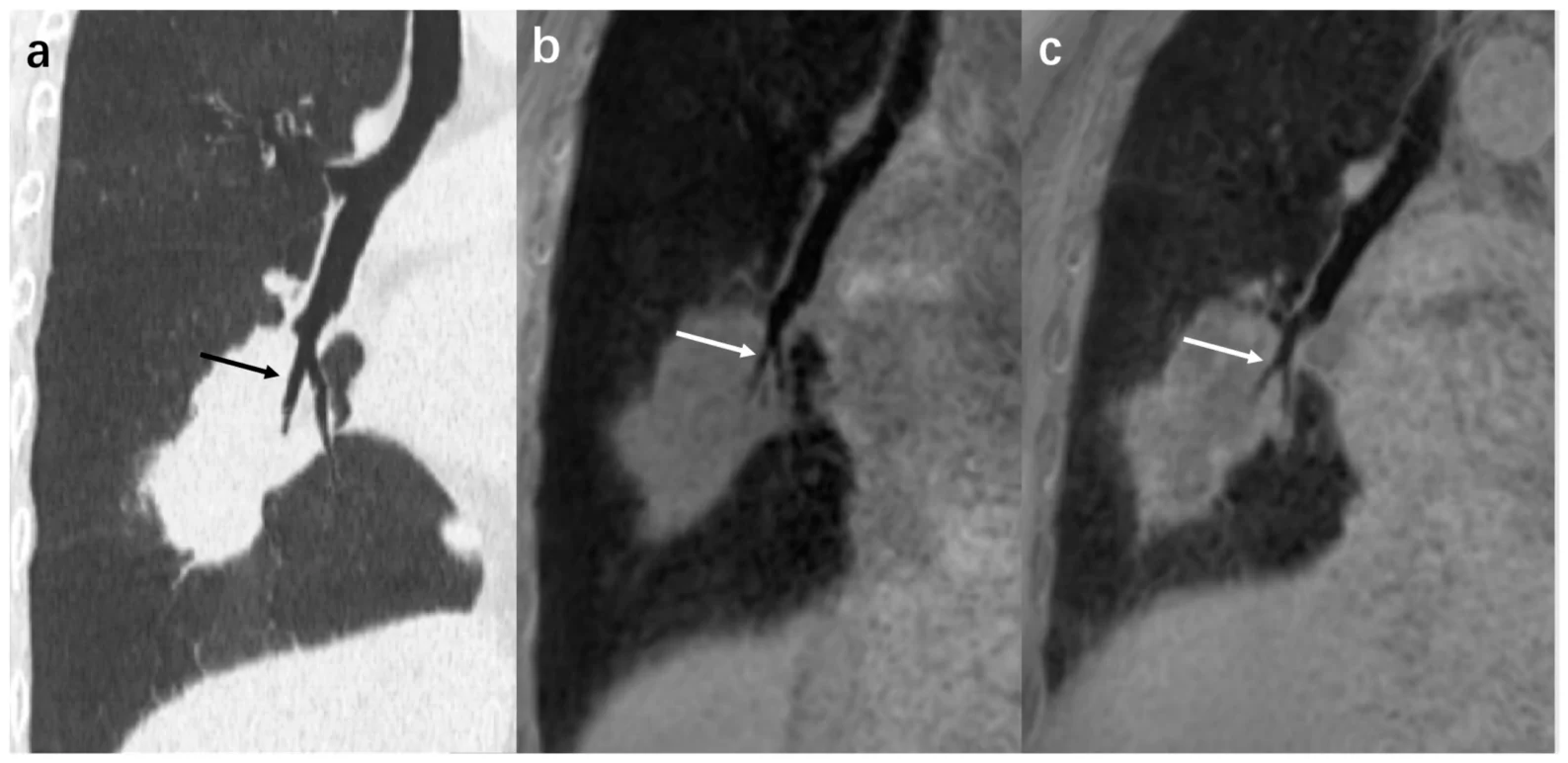
A patient with malignant lung tumor in right lower lobe. The air-bronchogram sign (arrows) within the tumor was clearly shown on minimum intensity projection (MinIP) images of CT (**a**), UTEu (**b**), UTEe (**c**).

**Figure 6:**
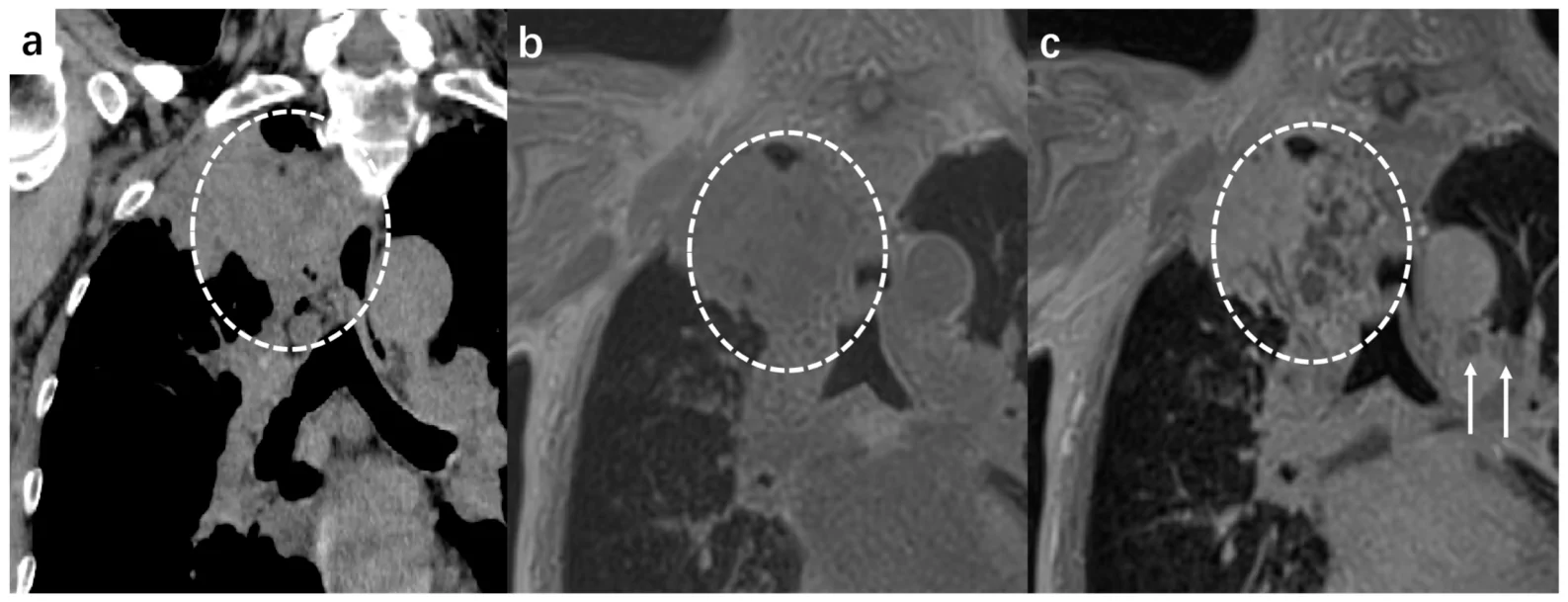
A patient with malignant tumor in the right upper lobe. The malignant tumor was clearly visible on mediastinal window CT (**a**), UTEu (**b**) and UTEe (**c**). The bubble-like necrosis within the tumor (white circle) was clearly shown on (**a**,**c**) but was obscure in (**b**) as inhomogeneous signal intensity; the range of necrosis was difficult to define. Moreover, necrosis of lymphatic nodes (arrows) in hilum was better depicted on (**c**) than on (**b**).

**Figure 7:**
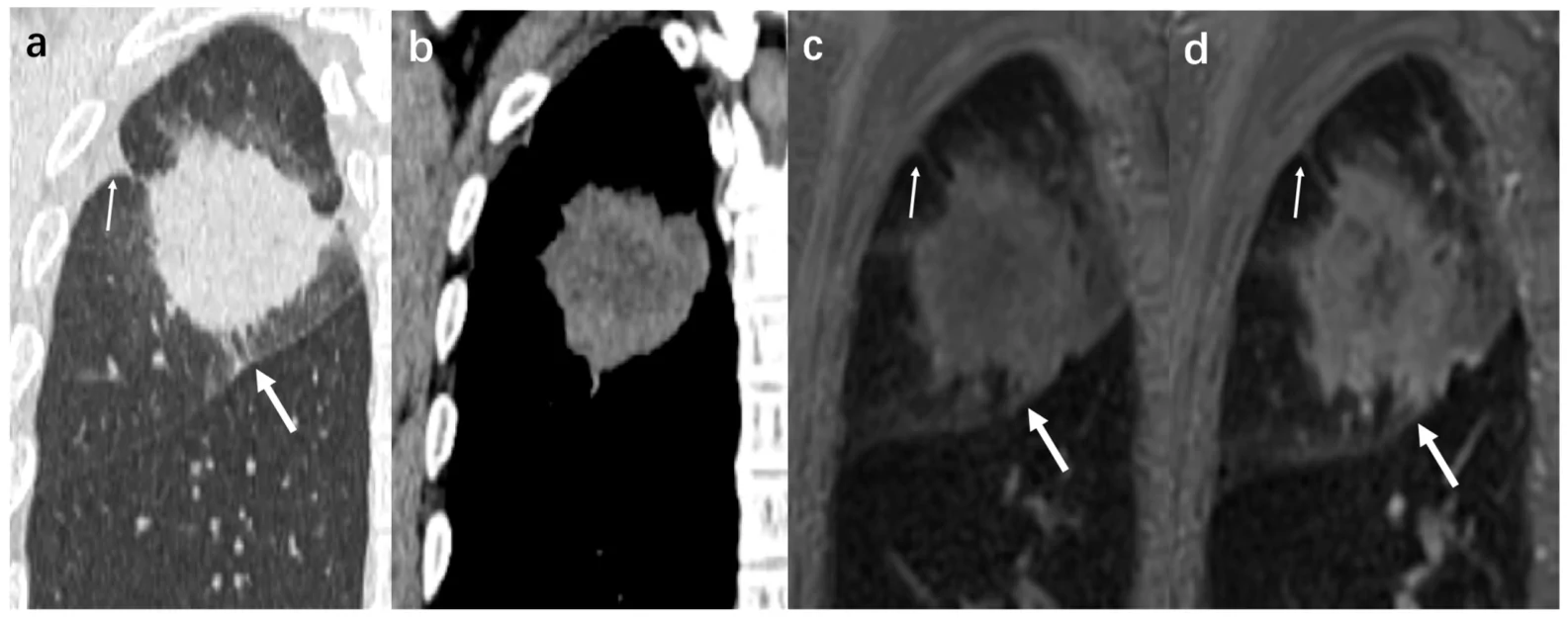
A patient with lung cancer in the right upper lobe. The coronal lung window (**a**) of CT showed the tumor with tiny spiculations, the nearby fissure (bold arrow) and pleura (arrow) were involved, and there was central hypo-intensity within the lesion on coronal mediastinal window (**b**) of CT. These findings were reliably visible on coronal UTEu and UTEe (**c**,**d**).

In field-strength-stratified end-to-end analyses, successful-characterization rates ranged from 55 to 71% for UTEe and 51–71% for UTEu derived from experienced reader panel, and 44–57% for UTEe and 42–57% for UTEu derived from inexperienced reader panel. The end-to-end characterization rates of pulmonary nodule morphological features were nearly overlapping between UTEe and UTEu at both 1.5 T (Figure 2A) and 3.0 T (Figure 2B), with the percentage-point differences (UTEe − UTEu, Figure 2C) clustering close to zero for most features. The representative images were shown in Figure 3, Figure 4, Figure 5, Figure 6 and Figure 7.

**Figure 8:**
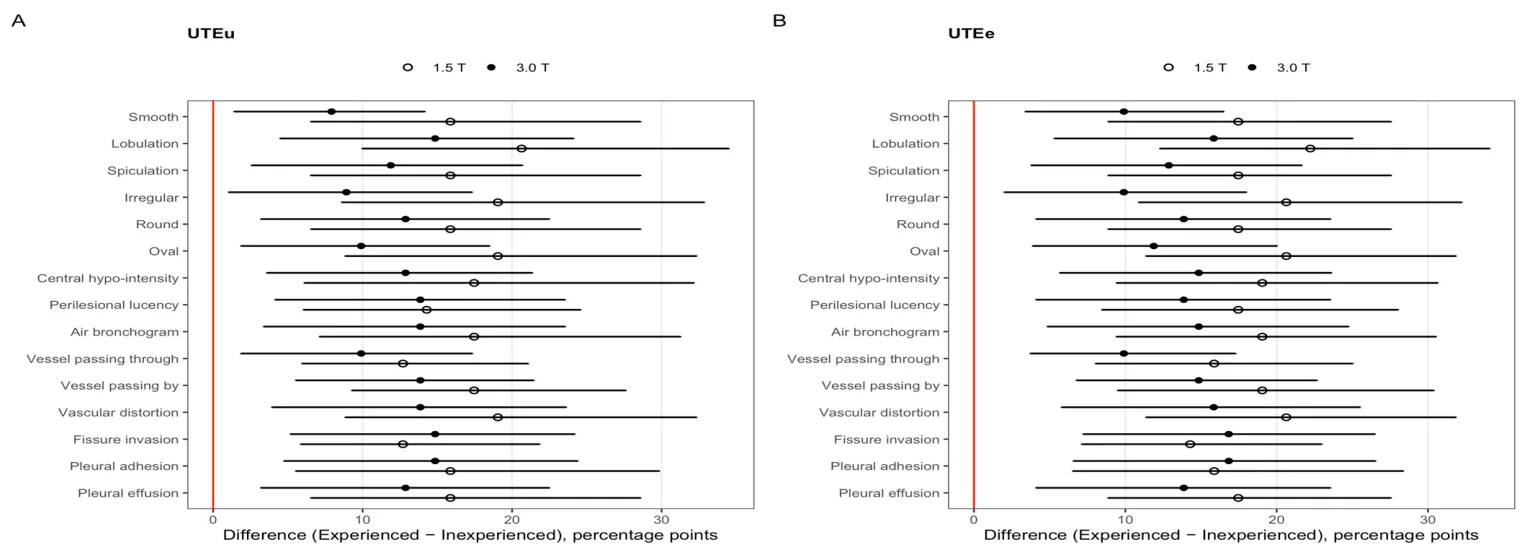
Differences in nodule morphological feature characterization between experienced and inexperienced reader panels. (**A**) UTEu at 1.5 T and 3.0 T. (**B**) UTEe at 1.5 T and 3.0 T. Open and filled circles represent 1.5 T and 3.0 T, respectively, with horizontal lines indicating confidence intervals. The red vertical line denotes no difference between experienced and inexperienced readers (0 percentage points).

Percentage-point differences (experienced minus inexperienced reader panels) for nodule feature characterization were positive across all evaluated signs for both UTEu (Figure 8A) and UTEe (Figure 8B) at 1.5 T and 3.0 T, indicating higher characterization rates by experienced panel (Figure 3). The magnitude of inter-reader panel differences was comparable between UTEu and UTEe, with most differences falling between 10 and 20 percentage points. In summary, inter-reader panel variability existed for both sequences, and the extent of panel-related performance gap was similar for UTEu and UTEe at both field strengths.

### 3.3. Conditional Morphology Performance

Among lesions visualized on both acquisitions, conditional morphology-level performance was evaluated using the same lesion set for UTEu and UTEe. The experienced-panel analysis included 113 common-visible lesions (42 at 1.5 T and 71 at 3.0 T; Figure 1). No statistically detectable UTEu–UTEe differences were demonstrated for most evaluated features. Conditional accuracy was relatively high for several features; however, these estimates describe morphology interpretation only after successful visualization and should be interpreted together with the corresponding end-to-end successful-characterization results. Detailed feature-level estimates, denominators, and 95% CIs are provided in Supplementary Tables S1 and S2.

### 3.4. Evaluation of Nodular Size Measurement

Significant differences in maximum lesion diameter were observed among UTEu, UTEe, and CT at both 1.5 T and 3.0 T (*p* < 0.001, Table 2). Pairwise comparisons showed that UTEu measurements differed significantly from CT at both field strengths, whereas no significant difference was observed between UTEe and CT at either field strength.

Negative ΔAE (AE_UTEe−AE_UTEu) indicated smaller absolute error for UTEe than UTEu for both 1.5 T and 3.0 T cohorts (Table 2). Scatter plots showed the lesion size measurements obtained by CT, UTEu and UTEe for each lesion at 1.5 T and 3.0 T (Supplementary Figure S1). Bland–Altman analysis demonstrated that both UTE sequences tended to underestimate lesion diameter relative to CT, although the magnitude of the mean bias was numerically smaller for UTEe than for UTEu at both field strengths (Supplementary Figure S2, Table 3). Linear regression analyses showed that the absolute measurement differences between CT and both UTE sequences increased significantly with lesion size. Lesion size explained 15.2% and 16.6% of the variance in |CT-UTEu| and |CT-UTEe|, respectively (Table 4). Durbin–Watson statistics were close to two for both models, indicating no substantial autocorrelation of the residuals.

### 3.5. Quantitative Image Characteristics of UTEu and UTEe According to Field-Strength Cohort

At 1.5 T, mean CNR was 13.6 ± 5.8 for UTEu and 16.4 ± 4.5 for UTEe, with no statistically significant between-acquisition difference (*p* = 0.36). At 3.0 T, the corresponding values were 19.1 ± 8.6 and 30.4 ± 16.3, respectively, with a statistically significant difference between the sequential acquisitions (*p* < 0.001). Because the 1.5 T and 3.0 T cohorts consisted of different patients examined on different scanner systems, between-field-strength comparisons were considered exploratory.

For edge sharpness, a small difference (mean ± SD, 0.06 ± 0.03) between UTEe and UTEu was observed in the 1.5 T cohort (*p* = 0.04), whereas no significant difference was observed in the 3.0 T cohort (*p* = 0.88) (mean ± SD, 0.00 ± 0.02).

### 3.6. Comparison and Agreement Between the Two Consensus Reader Panels

In the present dataset, the experienced consensus panel showed higher observed accuracy for morphological interpretation than the inexperienced consensus panel at both field strengths. Agreement between the final consensus assessments of the two panels was substantial to excellent. These findings describe differences and agreement between the two fixed panels and should not be interpreted as evidence of an independent effect of reader experience. For lesion visualization, agreement between the two consensus panels was substantial, with Cohen’s kappa values of 0.67 (95% CI: 0.49–0.85) for UTEu at 1.5 T; 0.64 (95% CI: 0.47–0.82) for UTEe at 1.5 T; 0.73 (95% CI: 0.59–0.86) for UTEu at 3.0 T; and 0.70 (95% CI: 0.57–0.84) for UTEe at 3.0 T (Supplementary Table S3).

### 3.7. Exploratory Analyses According to Field Strength

Because the magnetic field strength was confounded with several factors, the mixed-effect model does not eliminate confounding between two non-randomized patient groups. The data showed that no statistically significant evidence was found that field-strength group was associated with the evaluated lesion-level characteristics after accounting for within-patient clustering of multiple lesions (Table 5 provided the patient-level and lesion-level characteristics between the field-strength groups).

## 4. Discussion

For all lesions identified on CT, the morphological characterization of pulmonary nodules by spiral-UTE was limited by incomplete lesion visibility. We observed no significant morphological superiority of the delayed post-contrast acquisition over the preceding pre-contrast acquisition under the specific clinical protocol in our study.

For the evaluated morphological features, no statistically detectable differences in sensitivity, specificity, or accuracy were demonstrated between UTEu and UTEe within the analyzed sample with limited UTE visibility. However, these findings should not be interpreted as demonstrating equivalence or non-inferiority. The present study was not designed as an equivalence or non-inferiority trial; no clinically acceptable margin was prespecified. In addition, several morphological findings were represented by relatively few positive cases, limiting the robustness and clinical interpretability of the corresponding estimates. Many prior UTE studies [12,13,14,27,32,33,36] have used varying lesion-size criteria, acquisition protocols and field strengths and have primarily focused on CT-referenced pulmonary nodule detection, with reported detection rates ranging from 67.0% to 97.8%, broadly encompassing the 70.7% (116/164) visualization rate observed in our experienced consensus panel. In contrast, Wielpütz et al. [16] and Wang et al. [15] specifically evaluated morphological features on UTE relative to CT. Wielpütz et al. [16] reported 54.9–70.6% sensitivity for lobulation and 48.0–61.5% for spiculation, whereas a more recent respiratory-gated double-echo UTE study [37] reported detection rates of 96.9% and 81.8% for lobulation and spiculation, respectively. In the present study, paired UTEu–UTEe differences in end-to-end successful-characterization rates were close to zero for many morphological features, although larger numerical differences and wider confidence intervals were observed for some less frequent findings. In the complementary common-visible analysis, conditional sensitivity, specificity, and accuracy likewise showed no statistically detectable between-acquisition differences for most evaluated features.

Aligning with these findings [15,16], our spiral-UTE achieved maximum 79–100% sensitivity (except peripheral lucency) and 88–100% accuracy, with no statistically detectable UTEu–UTEe difference in this sample, from the exploratory analysis evaluating conditional morphology-level performance among UTE-visible lesions by experienced reader panel in current study. Notably, spiculation—a key malignant marker—showed high accuracy (92–100%). Peripheral lucency, however, had low sensitivity (33–57%), likely due to MRI’s limited ability to distinguish small lucencies from background lung. These findings indicate that, once a lesion is successfully visualized on spiral-UTE, several clinically relevant morphological features can be characterized with relatively high conditional accuracy relative to CT. Calcified nodules were excluded from current analysis, as MRI cannot reliably differentiate low-signal calcium from lung tissue.

Some numerical differences were observed in the depiction of central hypointense or necrotic components on delayed UTEe. However, because contrast condition was confounded with acquisition order and elapsed examination time, the mechanism and clinical significance of these observations cannot be determined from the present study and require prospective validation. Additionally, the benefits of UTEe should be weighed against the additional cost and potential risks associated with gadobutrol administration, including hypersensitivity reactions, gadolinium retention of uncertain clinical significance, and the very low but potential risk of nephrogenic systemic fibrosis (NSF) in patients with severe renal dysfunction, particularly those with acute kidney injury or with an eGFR < 30 mL/min/1.73 m^2^.

End-to-end and conditional analyses offer complementary insights into spiral-UTE morphology assessment. The end-to-end successful-characterization rate reflects both lesion visualization and morphology correctness and thus mirrors clinically achievable performance across all eligible CT-detected lesions. In contrast, conditional sensitivity, specificity, and accuracy evaluate morphology interpretation only after a lesion has been successfully visualized on both acquisitions.

The distinction between these two levels of performance is clinically important. Although conditional accuracy was high for several morphological features, lesion nonvisualization substantially reduced end-to-end successful-characterization rates. In the experienced consensus panel, 27.4% (45/164) of CT-detected lesions were visible on neither acquisition; in the inexperienced panel, this corresponding proportion was 43.3% (71/164). Accordingly, conditional morphology estimates should not be interpreted in isolation as overall spiral-UTE performance.

Paired visibility was highly concordant but not identical between acquisitions. In the experienced panel, three lesions were visualized only on UTEu and three only on UTEe; in the inexperienced panel, two lesions were visualized only on UTEu and none only on UTEe. Nevertheless, incorporating all lesions in the end-to-end analysis ensures that such sequence-specific visibility differences are retained rather than removed by restriction to the common-visible subset.

Both UTEu and UTEe underestimated nodule size by ~1 mm vs. CT, consistent with prior UTE-CT comparisons—respiratory-gated radial UTE, for instance, reported underestimation of ~1–2 mm [16,37]. This discrepancy may stem from the following key factors: (a) inherent tendency of MRI to smooth nodular margins [38]; (b) free-breathing-induced blurring of nodule boundaries in spiral-UTE sequences; (c) unavoidable biases from different respiratory modes (CT: end-inspiratory; spiral-UTE: free-breathing) and acquisition orientations (CT: axial; spiral-UTE: coronal) [39]; and (d) motion from both respiration and heartbeat. In the current study, UTEe showed a numerically smaller measurement bias relative to CT than UTEu. However, because UTEu was invariably acquired first and UTEe was obtained later during the delayed post-contrast phase, the observed difference cannot be attributed specifically to gadolinium-enhanced lesion delineation [40,41] and may also reflect acquisition order, elapsed examination time, respiratory or motion variability, or other time-dependent factors. A modest association was observed between lesion size and measurement discrepancy, although lesion size explained only a limited proportion of the overall variability. Overall, the difference in measurement error between UTEu and UTEe was small, and its magnitude was limited relative to the acquired voxel size (1.6 × 1.6 × 1.6 mm^3^). Therefore, the observed numerical difference should be interpreted cautiously, and its clinical relevance remains uncertain. Further validation in larger cohorts and study designs capable of separating contrast effects from acquisition-order and time-related effects is warranted.

The 1.5 T and 3.0 T examinations were performed in separate, non-randomized patient cohorts using different scanner systems; therefore, field-strength comparisons in the present study were considered exploratory rather than causal. To avoid conflating the primary UTEu–UTEe comparison with between-cohort differences, morphological analyses were conducted separately within the 1.5 T and 3.0 T cohorts. Although exploratory analyses suggested some differences between field-strength groups, these findings should not be interpreted as evidence of an independent field-strength effect. Magnetic field strength was inherently confounded with scanner platform and between-cohort differences, including patient characteristics, lesion size and composition, and other measured or unmeasured factors. Mixed-effect models accounted for clustering of multiple lesions within patients and adjusted for available measured covariates, but they could not remove residual confounding arising from the non-randomized, between-subject design or separate scanner systems. Accordingly, the present data do not permit isolation of the independent contribution of magnetic field strength to UTE performance. A paired or prospectively controlled design would be required to more rigorously evaluate field-strength effects. The observed between-cohort differences should therefore be regarded as exploratory and hypothesis-generating.

Previous studies have demonstrated the feasibility of UTE MRI for pulmonary nodule detection and characterization, although differences in acquisition trajectories, field strengths, lesion-size thresholds, and evaluation criteria have resulted in variable diagnostic performance. In particular, Wielpütz et al. [16] and Wang et al. [15] evaluated unenhanced UTE MRI against CT for several morphological characteristics, including lesion size and margin features, providing evidence that UTE can depict clinically relevant pulmonary nodule morphology. Our previously published study [29] and the current study share the following several methodological features: both were prospective studies in oncology populations, used free-breathing stack-of-spirals UTE MRI with both unenhanced (UTEu) and delayed post-contrast (UTEe) acquisitions, and used chest CT as the reference standard. However, the two studies addressed different diagnostic questions. The previous study primarily evaluated pulmonary nodule visualization/detection, whereas the present study extends this work to morphology-level assessment by explicitly integrating lesion visualization with morphology correctness across all CT-detected lesions and by additionally examining conditional morphology performance after successful visualization.

Importantly, there was no overlap in enrolled patients, CT or MRI examinations, source imaging datasets, or prospectively collected data between the two studies. The previous study [29] enrolled patients between January 2022 and February 2023 and was conducted exclusively at 1.5 T, whereas the present cohort was prospectively recruited between May 2024 and August 2026 and included separate cohorts examined at 1.5 and 3.0 T. Thus, the present investigation represents a methodologically related but independent extension of our previous work rather than a reanalysis of the previously published dataset. The previous study reported closely matched observed nodule-detection rates between UTEu and UTEe, whereas the present study evaluates a broader spectrum of morphology-level features, including attenuation type, margin characteristics, lobulation, spiculation, shape, internal features, air-bronchogram, vascular relationships, fissural involvement, and pleural relationships. The present study also included a descriptive comparison between two fixed consensus panels with different experience profiles. The principal extension of the present study is its detailed evaluation of pulmonary nodule morphology beyond lesion detection, together with explicit separation of lesion visualization, end-to-end successful characterization, and conditional morphology performance after successful visualization.

Delayed post-contrast UTEe showed higher observed lesion CNR in some analyses; however, this was not consistently accompanied by statistically detectable differences in morphology-level performance. Some numerical differences were also observed for individual internal features, including central hypointensity. Because UTEu was invariably acquired first and UTEe was obtained later during the delayed post-contrast phase, these observations cannot be attributed specifically to gadolinium administration and may also reflect acquisition order, elapsed examination time, respiratory or motion variability, or other time-dependent factors. Therefore, the present findings characterize the observed performance of sequential pre-contrast and delayed post-contrast spiral-UTE within the clinical workflow used in this study, rather than the isolated effect of gadolinium. They do not establish whether gadolinium administration is necessary or unnecessary for pulmonary nodule morphology assessment.

CT was used as the morphological reference standard, and differences between CT and MRI acquisition conditions should also be considered when interpreting the results. Although the chest CT examinations followed a standardized clinical protocol with broadly consistent reconstruction settings, residual variability related to scanner platform, automatic exposure modulation, patient body habitus, and other acquisition factors may have affected CT image quality. In addition, CT was acquired during end-inspiratory breath-hold, whereas spiral-UTE was performed during free breathing, introducing unavoidable differences in respiratory state and lesion appearance. CT-derived signal intensity or noise measurements were not incorporated into the quantitative MRI analyses; CT served only as anatomical and morphological reference.

This study has several limitations.

First, the conditional morphology analysis was restricted to lesions visualized on both UTE acquisitions and its formal interpretation as a conditional estimand was not prospectively specified in a registered protocol or statistical analysis plan; it should therefore be regarded as a post hoc complementary analysis. To address the associated selection concern, we additionally evaluated end-to-end successful characterization across all 164 CT-detected eligible lesions, with nonvisualized lesions designated not characterizable and unsuccessful for the composite outcome. Nevertheless, this end-to-end measure is a composite of lesion visibility and morphology correctness rather than a conventional feature-specific sensitivity or specificity metric. Moreover, the substantial proportion of nonvisualized lesions remains an important limitation of clinically achievable spiral-UTE performance.

Second, the acquisition design did not isolate the independent effect of gadolinium administration. UTEu was invariably acquired first, whereas UTEe was obtained approximately 10–15 min after contrast administration and after completion of the clinically indicated head or abdominal MRI sequences; moreover, the post-injection interval was not strictly standardized. Consequently, observed differences, or the absence of differences, between UTEu and UTEe may have been influenced by acquisition order, elapsed examination time, respiratory or motion variability, patient fatigue, physiologic variation, or delayed enhancement kinetics. The findings should therefore be interpreted as a comparison between sequential pre-contrast and delayed post-contrast acquisitions under the clinical workflow used in this study rather than as evidence of an isolated gadolinium effect.

Third, the study was not prospectively designed within an equivalence or non-inferiority framework, and no clinically acceptable margin for differences in morphological performance was prespecified. Accordingly, the absence of statistically detectable UTEu–UTEe differences cannot be interpreted as evidence of equivalence or non-inferiority. Several morphological findings were represented by relatively few positive cases, limiting the robustness and clinical interpretability of the corresponding estimates; for some less frequent findings, the confidence intervals of the paired differences were also wide.

Fourth, the reader-study design does not permit a generalizable inference regarding an independent effect of radiologist experience. The experienced and inexperienced conditions each consisted of one fixed pair of radiologists, and final assessments were based on within-panel consensus. Thus, experience level was confounded with reader identity and potentially with the consensus process. Because formal individual pre-consensus reader-level assessments were not generated or recorded, a multireader analysis treating reader as a random effect was not feasible. The observed differences should therefore be interpreted as differences between these two specific consensus panels rather than as evidence that reader experience itself independently determines spiral-UTE performance.

Fifth, the 1.5 T and 3.0 T examinations were performed in separate, non-randomized patient cohorts using different scanner systems. Although mixed-effect analyses accounted for clustering of multiple lesions within patients and adjusted for available measured covariates, residual and unmeasured between-cohort confounding could not be eliminated. Because field strength was also confounded with scanner platform, the independent contribution of magnetic field strength could not be isolated. Accordingly, field-strength comparisons should be regarded as exploratory and hypothesis-generating.

Sixth, several aspects of the study population and reference-standard comparison may limit generalizability. The cohort predominantly consisted of patients with known malignancies undergoing evaluation for suspected metastases, and nodules < 5 mm and calcified nodules were excluded. Pathological confirmation was unavailable for the enrolled nodules/masses, precluding assessment of the ability of spiral-UTE to distinguish benign from malignant lesions. In addition, CT and MRI were acquired under different conditions, including end-inspiratory breath-hold CT versus free-breathing UTE, and differences in patient positioning, acquisition orientation, and possible coil-related compression may have affected direct comparison of lesion appearance and position. Although the CT-to-MRI interval was limited to ≤4 days, interval-related changes cannot be completely excluded.

Seventh, several evaluated morphological signs were derived from conventional CT interpretation, whereas CT and UTE MRI differ fundamentally in image-formation mechanisms and tissue contrast. Consequently, features such as ground-glass opacity, air-bronchogram, and vascular distortion may not have identical visual manifestations across the two modalities, which should be considered when interpreting MRI-CT agreement.

Eighth, because SPIRiT-based parallel imaging and reconstruction may produce spatially varying and potentially non-Gaussian noise, the airway signal-intensity SD used in the CNR calculation represents an empirical measure of signal variability rather than a rigorous estimate of the underlying noise distribution. Averaging measurements from the trachea and bilateral main bronchi was intended to reduce regional variability, but this limitation should be considered when interpreting the CNR results.

Ninth, edge sharpness was manually measured by a single radiologist. Although each measurement was repeated 2–3 times and averaged to reduce within-procedure measurement variability, no formal intra-observer assessment across independent reading sessions or interobserver reproducibility analysis was performed. Therefore, the reproducibility of this measure remains uncertain, and the edge-sharpness findings should be regarded as exploratory. Edge sharpness was a secondary exploratory quantitative endpoint and did not contribute to the primary end-to-end successful-characterization analysis or the complementary conditional morphology analysis. Similarly, lesion-size measurements were obtained by consensus within each reader panel, and individual reader-level repeatability of size measurements was not prospectively assessed.

## 5. Conclusions

Across all CT-detected eligible lesions, clinically achievable morphological characterization on spiral-UTE was limited by incomplete lesion visualization. End-to-end successful-characterization analyses did not demonstrate a statistically detectable UTEu–UTEe difference for most evaluated morphological features, whereas the complementary common-visible analysis showed relatively high conditional morphology-level performance for several features after successful lesion visualization. Because contrast condition was confounded with acquisition order and post-injection timing, and no equivalence or non-inferiority framework was prespecified, these findings neither isolate an independent gadolinium effect nor establish equivalent performance between UTEu and UTEe.

## Figures and Tables

**Table 1:** Patient and MR examination characteristics.

Characteristic	Value
Total number of patients	61
Male	35 (57.4%)
Female	26 (42.6%)
Age, years	58.4 ± 10.4
Age range, years	19~74
Total number of pulmonary nodules/masses	164
1.5 T group	27 (44.3%)
3.0 T group	34 (55.7%)
Patients with a single pulmonary nodule/mass	25 (41.0%)
Patients with multiple pulmonary nodules/masses	36 (59.0%)
Interval between CT and MRI, days	0.75 ± 1.03
Range of CT-MRI interval, days	0~4
Successful completion of both UTEu and UTEe scans	61 (100%)
Allergic reactions	0 (0%)

Note: Data are presented as number (%), mean ± standard deviation, or range, *n* (%) as appropriate.

**Table 2:** Comparison of maximum lesion diameter measured using CT, unenhanced UTE (UTEu), and contrast-enhanced UTE (UTEe).

Field Strength	CT (mm)	UTEu (mm)	UTEe (mm)	*p* Value				ΔAE * = AE_UTEe − AE_UTEu	
				Overall	UTEu vs. CT	UTEe vs. CT	UTEu vs. UTEe	Mean ± SD	95% CI
1.5 T	14.9 (8.7–27.1)	14.2 (8.1–24.7)	14.5 (8.1–27.1)	0.007	0.010	0.422	0.420	−0.252 ± 0.127	−0.511~0.008
3.0 T	13.2 (8.1–26.2)	12.6 (7.2–24.6)	13.0 (7.2–25.7)	<0.001	<0.001	0.624	<0.001	−0.586 ± 0.117	−0.825–0.347

Note: Data are presented as median (IQR) or mean ± SD; pairwise *p* values were adjusted using the Bonferroni correction; *: a lesion-wise analysis of absolute measurement error (AE) using CT as the reference standard was performed.

**Table 3:** Bland–Altman bias in maximum lesion diameter measurements relative to CT.

Field Strength	Comparison	Mean Bias, mm	95% Limits of Agreement (95% LoA)	Direction of Bias
1.5 T	UTEu versus CT	−0.73	−2.79~4.24	UTEu underestimated lesion diameter
1.5 T	UTEe versus CT	−0.36	−2.33~3.06	UTEe underestimated lesion diameter
3.0 T	UTEu versus CT	−1.08	−2.59~4.75	UTEu underestimated lesion diameter
3.0 T	UTEe versus CT	−0.69	−3.46~4.84	UTEe underestimated lesion diameter

Note: UTEu, unenhanced spiral-UTE; UTEe, contrast-enhanced spiral-UTE.

**Table 4:** Linear regression analysis of the association between lesion size and absolute CT–UTE measurement difference.

Dependent Variable	R^2^	Adjusted R^2^	Unstandardized Co-Efficient, B	Standardized Coefficient, β	Slope *p* Value	Overall Model *p* Value
|CT − UTEu|	0.152	0.144	0.035	0.390	<0.001	<0.001
|CT − UTEe|	0.166	0.158	0.038	0.407	<0.001	<0.001

Note: UTEu, unenhanced spiral-UTE; UTEe, contrast-enhanced spiral-UTE.

**Table 5:** Comparison of patient- and lesion-level characteristics between the 1.5-T and 3.0-T groups.

Characteristics	1.5 T Group	3.0 T Group	*p* Value
Patient-level			
Number of patients	27	34	-
Age, years	56.89 ± 12.31	59.56 ± 8.52	0.503 ^1^
Male sex, *n* (%)	13 (48.15%)	22 (64.71%)	1.000 ^2^
CT-to-MRI interval, days	0.70 ± 0.91	0.74 ± 0.90	0.911 ^3^
Lesions per patient, *n*	2 (1–3)	2 (1–4)	0.570 ^3^
Multiple lesions (≥2), *n* (%)	18 (66.67%)	18 (52.94%)	0.251 ^2^
Primary malignancy, *n* (%)	18 (66.67%)	25 (73.53%)	0.080 ^2^
Lesion-level			
Lesion number, *n*	63	101	-
CT maximum diameter, mm	25.33 ± 3.52	23.71 ± 2.93	0.726 ^4^
Adjacent to diaphragm, *n* (%)	21 (33.33%)	41 (40.59%)	0.349 ^5^
Adjacent to heart, *n* (%)	20 (31.75%)	21 (20.79%)	0.126 ^5^
Adjacent to pleura, *n* (%)	19 (30.16%)	25 (24.75%)	0.449 ^5^
Lesion type (solid, GGO, mGGO)			0.126 ^6^
Solid, *n* (%)	38 (60.32%)	82 (81.19%)	-
Pure GGO, *n* (%)	13 (20.63%)	12 (11.88%)	-
Mixed GGO, *n* (%)	12 (19.05%)	7 (6.93%)	-

Note: Statistical tests were selected according to variable type, distribution, and clustering structure. Approximately normally distributed continuous patient-level variables were compared using the independent-samples *t* test, whereas non-normally distributed continuous variables were compared using the Mann–Whitney U test. Categorical patient-level variables were analyzed using the chi-square test or Fisher’s exact test, as appropriate. For lesion-level analyses, mixed-effect models with patient-specific random intercepts were used to account for clustering of multiple lesions within the same patient; linear, logistic, or multinomial mixed-effect models were selected according to whether the outcome was continuous, binary, or multinomial, respectively. ^1^ from independent-samples *t* test; ^2^ from chi-square test/Fisher’s exact test; ^3^ from Mann–Whitney U test; ^4^ from mixed linear-effect model with a patient-specific random intercept to account for the clustering of lesions within patients; ^5^ from mixed-effect logistic regression model with field-strength group as a fixed effect and a patient-specific random intercept to account for clustering of lesions within patients; ^6^ from mixed-effect multinomial logistic regression model, with field-strength group as a fixed effect and a patient-specific random intercept to account for clustering of lesions within patients.

## Data Availability

All data generated or analyzed during this study are included in this published article and its Supplementary Information Files.

## Supplementary Materials

The following supporting information can be downloaded at https://www.mdpi.com/article/10.3390/diagnostics16193170/s1, Figure S1: Scatter plots showing lesion size measurements obtained by CT, UTEu, and UTEe for each sample at 1.5 T and 3 T. The x-axis represents the sample number, and the y-axis represents the measured lesion size.; Figure S2: Bland-Altman analyses of UTEu and UTEe with CT for maximum nodule size depiction at 1.5 T and 3 T; Table S1: Conditional morphology-level performance among UTE-visible lesions by experienced reader panel at 1.5 T; Table S2: Conditional morphology-level performance among UTE-visible lesions by experienced reader panel at 3.0 T; Table S3: Inter-panel agreement for nodule morphologic depiction for spiral-UTE at 1.5 T and 3.0 T.

## Abbreviation

The following abbreviations are used in this manuscript:

UTE	ultrashort TE
SPIRiT	iterative self-consistent parallel imaging reconstruction
TR	repetition time
TE	echo time
FOV	field of view
TA	acquisition time
GGO	ground glass opacity
CNR	contrast-to-noise ratio

## References

[1] Akazawa Y., Yoshikawa A., Kanazu M., Yano Y., Yamaguchi T., Mori M. (2022). Non-small cell lung cancer with tumor proportion score > 90% could increase the risk of severe immune-related adverse events in first-line treatments with immune checkpoint inhibitors: A retrospective single-center study. Thorac. Cancer.

[2] Bray F., Laversanne M., Sung H., Ferlay J., Siegel R.L., Soerjomataram I., Jemal A. (2024). Global cancer statistics 2022: GLOBOCAN estimates of incidence and mortality worldwide for 36 cancers in 185 countries. CA Cancer J. Clin..

[3] Thai A.A., Solomon B.J., Sequist L.V., Gainor J.F., Heist R.S. (2021). Lung cancer. Lancet.

[4] Osarogiagbon R.U., Liao W., Faris N.R., Meadows-Taylor M., Fehnel C., Lane J., Williams S.C., Patel A.A., Akinbobola O.A., Pacheco A. (2022). Lung Cancer Diagnosed Through Screening, Lung Nodule, and Neither Program: A Prospective Observational Study of the Detecting Early Lung Cancer (DELUGE) in the Mississippi Delta Cohort. J. Clin. Oncol..

[5] Lee H.J., Goo J.M., Lee C.H., Park C.M., Kim K.G., Park E.-A., Lee H.Y. (2009). Predictive CT findings of malignancy in ground-glass nodules on thin-section chest CT: The effects on radiologist performance. Eur. Radiol..

[6] Zhang R., Tian P., Chen B., Zhou Y., Li W. (2020). Predicting Lung Cancer Risk of Incidental Solid and Subsolid Pulmonary Nodules in Different Sizes. Cancer Manag. Res..

[7] Lee S.M., Park C.M., Goo J.M., Lee H.-J., Wi J.Y., Kang C.H. (2013). Invasive pulmonary adenocarcinomas versus preinvasive lesions appearing as ground-glass nodules: Differentiation by using CT features. Radiology.

[8] Lim H.-J., Ahn S., Lee K.S., Han J., Shim Y.M., Woo S., Kim J.-H., Yie M., Lee H.Y., Yi C.A. (2013). Persistent pure ground-glass opacity lung nodules ≥ 10 mm in diameter at CT scan: Histopathologic comparisons and prognostic implications. Chest.

[9] Erdogdu E., Ozkan B., Duman S., Agkoc M., Erturk S.M., Kara M., Toker A. (2022). Predictors of malignancy in patients with solitary pulmonary nodules undergoing pulmonary resection. Clin. Respir. J..

[10] Pearce M.S., Salotti J.A., Little M.P., McHugh K., Lee C., Kim K.P., Howe N.L., Ronckers C.M., Rajaraman P., Sir Craft A.W. (2012). Radiation exposure from CT scans in childhood and subsequent risk of leukaemia and brain tumours: A retrospective cohort study. Lancet.

[11] Brenner D.J., Hall E.J. (2012). Cancer Risks from CT Scans: Now We Have Data, What Next?. Radiology.

[12] Ohno Y., Takenaka D., Yoshikawa T., Yui M., Koyama H., Yamamoto K., Hamabuchi N., Shigemura C., Watanabe A., Ueda T. (2022). Efficacy of Ultrashort Echo Time Pulmonary MRI for Lung Nodule Detection and Lung-RADS Classification. Radiology.

[13] Ohno Y., Koyama H., Yoshikawa T., Kishida Y., Seki S., Takenaka D., Yui M., Miyazaki M., Sugimura K. (2017). Standard-, Reduced-, and No-Dose Thin-Section Radiologic Examinations: Comparison of Capability for Nodule Detection and Nodule Type Assessment in Patients Suspected of Having Pulmonary Nodules. Radiology.

[14] Ohno Y., Koyama H., Yoshikawa T., Seki S., Takenaka D., Yui M., Lu A., Miyazaki M., Sugimura K. (2016). Pulmonary high-resolution ultrashort TE MR imaging: Comparison with thin-section standard- and low-dose computed tomography for the assessment of pulmonary parenchyma diseases: Pulmonary MRI with UTE in Pulmonary Disease. J. Magn. Reson. Imaging.

[15] Wang F., Lin X., Lin C., Huang G., Li M., Zhu L. (2023). Ability of three-dimensional 3-Tesla ultrashort echo time magnetic resonance imaging to display the morphological characteristics of pulmonary nodules: A sensitivity analysis. Quant. Imaging Med. Surg..

[16] Wielpütz M.O., Lee H.Y., Koyama H., Yoshikawa T., Seki S., Kishida Y., Sakai Y., Kauczor H.-U., Sugimura K., Ohno Y. (2018). Morphologic Characterization of Pulmonary Nodules with Ultrashort TE MRI at 3T. Am. J. Roentgenol..

[17] John P., Mugler I., Fielden S.W., Meyer C.H., Altes T.A., Miller G.W., Stemmer A., Pfeuffer J., Kiefer B. Breath-hold UTE Lung Imaging using a Stack-of-Spirals Acquisition. Proceedings of the 23rd Annual Meeting of ISMRM.

[18] John P., Mugler I., Meyer C.H., Pfeuffer J., Stemmer A., Kiefer B. Accelerated Stack-of-Spirals Breath-hold UTE Lung Imaging. Proceedings of the 25th Annual Meeting of ISMRM.

[19] Dournes G., Yazbek J., Benhassen W., Benlala I., Blanchard E., Truchetet M.-E., Macey J., Berger P., Laurent F. (2018). 3D ultrashort echo time MRI of the lung using stack-of-spirals and spherical *k* -Space coverages: Evaluation in healthy volunteers and parenchymal diseases: Lung MRI with 3D UTE Spiral VIBE Sequence. J. Magn. Reson. Imaging.

[20] Cha M.J., Park H.J., Paek M.Y., Stemmer A., Lee E.S., Park S.B., Kim Y.S. (2018). Free-breathing ultrashort echo time lung magnetic resonance imaging using stack-of-spirals acquisition: A feasibility study in oncology patients. Magn. Reson. Imaging.

[21] Huang Y.-S., Niisato E., Su M.-Y.M., Benkert T., Hsu H.-H., Shih J.-Y., Chen J.-S., Chang Y.-C. (2021). Detecting small pulmonary nodules with spiral ultrashort echo time sequences in 1.5 T MRI. Magn. Reson. Mater. Phys. Biol. Med..

[22] Qian Y., Boada F.E. (2008). Acquisition-weighted stack of spirals for fast high-resolution three-dimensional ultra-short echo time MR imaging. Magn. Reson. Med..

[23] Kumar S., Rai R., Stemmer A., Josan S., Holloway L., Vinod S., Moses D., Liney G. (2017). Feasibility of free breathing Lung MRI for Radiotherapy using non-Cartesian k-space acquisition schemes. Br. J. Radiol..

[24] Cha M.J., Ahn H.S., Choi H., Park H.J., Benkert T., Pfeuffer J., Paek M.Y. (2021). Accelerated Stack-of-Spirals Free-Breathing Three-Dimensional Ultrashort Echo Time Lung Magnetic Resonance Imaging: A Feasibility Study in Patients with Breast Cancer. Front. Oncol..

[25] Dournes G., Grodzki D., Macey J., Girodet P.-O., Fayon M., Chateil J.-F., Montaudon M., Berger P., Laurent F. (2015). Quiet Submillimeter MR Imaging of the Lung Is Feasible with a PETRA Sequence at 1.5 T. Radiology.

[26] Johnson K.M., Fain S.B., Schiebler M.L., Nagle S. (2013). Optimized 3D ultrashort echo time pulmonary MRI. Magn. Reson. Med..

[27] Jiang Y., Pu D., Zhang X., Ren Z., Yu N. (2025). Comparison of diagnostic performance for pulmonary nodule detection between free-breathing spiral ultrashort echo time and free-breathing radial volumetric interpolated breath-hold examination. BMC Med. Imaging.

[28] Lustig M., Pauly J.M. (2010). SPIRiT: Iterative self-consistent parallel imaging reconstruction from arbitrary *k*-space. Magn. Reson. Med..

[29] Wu J.-W., Sun X., Li Z.-K., Meng X.-N., Li Q., Xiao A.-D., Yin T., Benkert T., Sun P., Lu H.-H. (2026). Performance of free-breathing contrast-enhanced and unenhanced stack-of-spirals ultrashort echo time MRI for lung follow-up in patients with malignancies at 1.5T. Quant. Imaging Med. Surg..

[30] Uhlig J., Al-Bourini O., Salgado R., Francone M., Vliegenthart R., Bremerich J., Lotz J., Gutberlet M. (2020). Gadolinium-based Contrast Agents for Cardiac MRI: Use of Linear and Macrocyclic Agents with Associated Safety Profile from 154,779 European Patients. Radiol. Cardiothorac. Imaging.

[31] Iyad N., S Ahmad M., Alkhatib S.G., Hjouj M. (2023). Gadolinium contrast agents- challenges and opportunities of a multidisciplinary approach: Literature review. Eur. J. Radiol. Open.

[32] Olthof S.-C., Reinert C., Nikolaou K., Pfannenberg C., Gatidis S., Benkert T., Küstner T., Krumm P. (2021). Detection of lung lesions in breath-hold VIBE and free-breathing Spiral VIBE MRI compared to CT. Insights Imaging.

[33] Burris N.S., Johnson K.M., Larson P.E.Z., Hope M.D., Nagle S.K., Behr S.C., Hope T.A. (2016). Detection of Small Pulmonary Nodules with Ultrashort Echo Time Sequences in Oncology Patients by Using a PET/MR System. Radiology.

[34] Larson A.C., Kellman P., Arai A., Hirsch G.A., McVeigh E., Li D., Simonetti O.P. (2005). Preliminary investigation of respiratory self-gating for free-breathing segmented cine MRI. Magn. Reson. Med..

[35] Fu Q., Liu D.-X., Zhang X.-Y., Deng X.-B., Zheng C.-S. (2021). Pointwise encoding time reduction with radial acquisition in subtraction-based magnetic resonance angiography to assess saccular unruptured intracranial aneurysms at 3 Tesla. Neuroradiology.

[36] Renz D.M., Herrmann K.-H., Kraemer M., Boettcher J., Waginger M., Krueger P.-C., Pfeil A., Streitparth F., Kentouche K., Gruhn B. (2022). Ultrashort echo time MRI of the lung in children and adolescents: Comparison with non-enhanced computed tomography and standard post-contrast T1w MRI sequences. Eur. Radiol..

[37] Li Q., Zhu L., Stackelberg O.V., Triphan S.M.F., Biederer J., Weinheimer O., Eichinger M., Vogelmeier C.F., Jörres R.A., Kauczor H.-U. (2023). MRI Compared with Low-Dose CT for Incidental Lung Nodule Detection in COPD: A Multicenter Trial. Radiol. Cardiothorac. Imaging.

[38] Meier-Schroers M., Homsi R., Gieseke J., Schild H.H., Thomas D. (2019). Lung cancer screening with MRI: Evaluation of MRI for lung cancer screening by comparison of LDCT- and MRI-derived Lung-RADS categories in the first two screening rounds. Eur. Radiol..

[39] Bankier A.A., O’Donnell C.R., Boiselle P.M. (2008). Quality Initiatives Respiratory Instructions for CT Examinations of the Lungs: A Hands-on Guide. RadioGraphics.

[40] Kono R., Fujimoto K., Terasaki H., Müller N.L., Kato S., Sadohara J., Hayabuchi N., Takamori S. (2007). Dynamic MRI of solitary pulmonary nodules: Comparison of enhancement patterns of malignant and benign small peripheral lung lesions. AJR Am. J. Roentgenol..

[41] Gückel C., Schnabel K., Deimling M., Steinbrich W. (1996). Solitary pulmonary nodules: MR evaluation of enhancement patterns with contrast-enhanced dynamic snapshot gradient-echo imaging. Radiology.

