# Supplementary material for: Comparison of Pulmonary Nodule Morphology on Pre-Contrast and Delayed Post-Contrast Stack-of-Spirals Ultrashort Echo Time MRI
Source: Diagnostics (Basel). 2026 Sep 29;16(19):3170. doi: 10.3390/diagnostics16193170 (PMC13664507; doi:10.3390/diagnostics16193170)
Supplement: Supplementary file 1 [file diagnostics-16-03170-s001.zip › diagnostics-4504924-supplementary.pdf]

Supplementary Materials

Figure S1. Scatter plots showing lesion size measurements obtained by CT, UTEu, and UTEe for each sample at 1.5T and 3T. The x-axis represents the sample number, and the y-axis represents the measured lesion size.

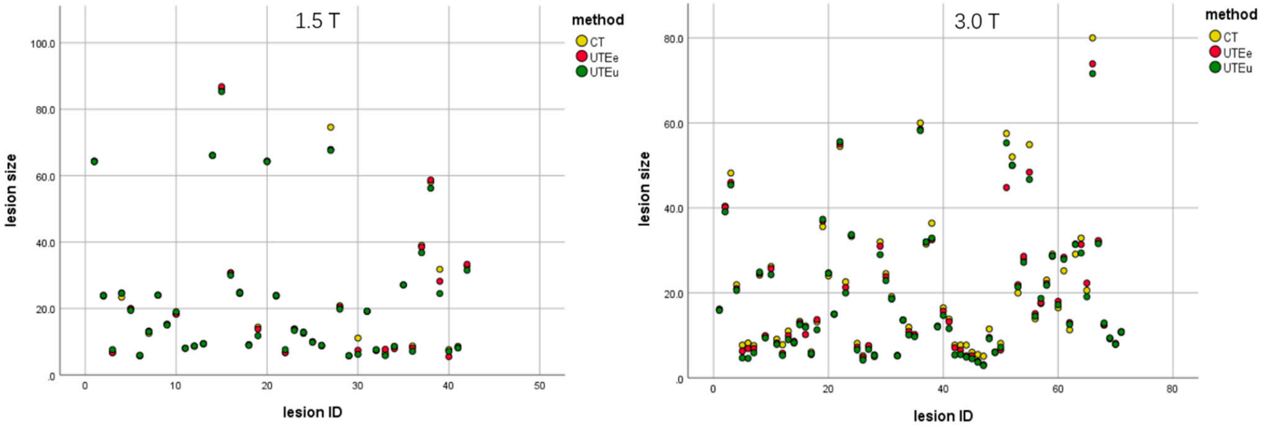

Figure S2. Bland-Altman analyses of UTEu and UTEe with CT for maximum nodule size depiction at 1.5T and 3T

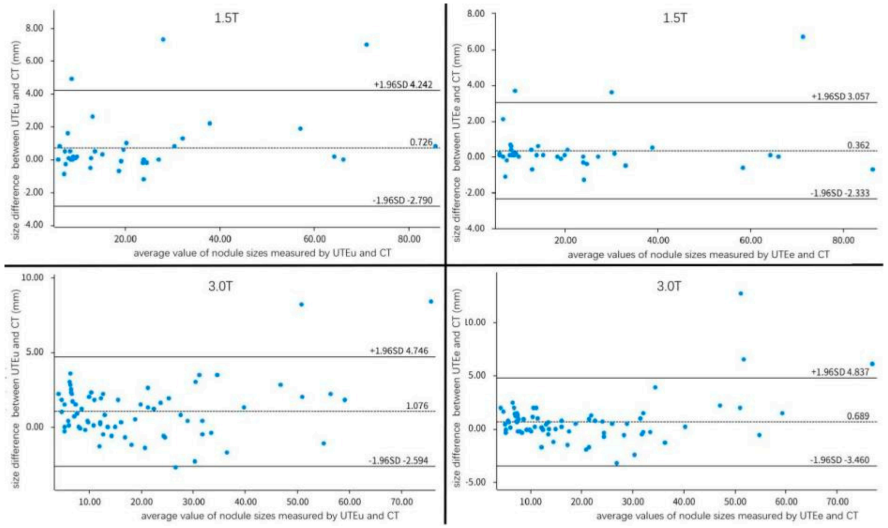

Table S1 Conditional morphology-level performance among UTE-visible lesions by experienced reader panel at 1.5T

Morphological features		Sensitivity		specificity		accuracy	
		UTEu	UTEe	UTEu	UTEe	UTEu	UTEe
Margin	smooth	1.00(15/15; 1.00,1.00)	1.00(15/15; 1.00,1.00)	1.00(27/27; 1.00,1.00)	1.00(27/27; 1.00,1.00)	1.00(42/42; 1.00,1.00)	1.00(42/42; 1.00,1.00)
	lobulation	1.00(22/22; 1.00,1.00)	1.00(22/22; 1.00,1.00)	1.00(20/20; 1.00,1.00)	1.00(20/20; 1.00,1.00)	1.00(42/42; 1.00,1.00)	1.00(42/42; 1.00,1.00)
	spiculation	1.00(14/14; 1.00,1.00)	1.00(14/14; 1.00,1.00)	1.00(28/28; 1.00,1.00)	1.00(28/28; 1.00,1.00)	1.00(42/42; 1.00,1.00)	1.00(42/42; 1.00,1.00)
	irregular	1.00(23/23; 1.00,1.00)	1.00(23/23; 1.00,1.00)	1.00(19/19; 1.00,1.00)	1.00(19/19; 1.00,1.00)	1.00(42/42; 1.00,1.00)	1.00(42/42; 1.00,1.00)
Dominant shape	round	1.00(6/6; 1.00,1.00)	1.00(6/6; 1.00,1.00)	1.00(36/36; 1.00,1.00)	1.00(36/36; 1.00,1.00)	1.00(42/42; 1.00,1.00)	1.00(42/42; 1.00,1.00)
	oval	0.96(24/25; 0.86,1.00)	0.96(24/25; 0.86,1.00)	1.00(17/17; 1.00,1.00)	1.00(17/17; 1.00,1.00)	0.98(41/42; 0.92,1.00)	0.98(41/42; 0.92,1.00)
Cavitation	Central hypo-intensity of lesion	0.92(11/12; 0.69,1.00)	1.00(12/12; 1.00,1.00)	0.97(29/30; 0.89,1.00)	0.97(29/30; 0.89,1.00)	0.95(40/42; 0.87,1.00)	0.98(41/42; 0.92,1.00)
	Perilesional lucency	0.33(1/3; 0.00,1.00)	0.33(1/3; 0.00,1.00)	1.00(39/39; 1.00,1.00)	1.00(39/39; 1.00,1.00)	0.95(40/42; 0.84,1.00)	0.95(40/42; 0.84,1.00)
	Air-bronchogram	0.92(12/13; 0.78,1.00)	0.92(12/13; 0.78,1.00)	1.00(29/29; 1.00,1.00)	1.00(29/29; 1.00,1.00)	0.98(41/42; 0.92,1.00)	0.98(41/42; 0.92,1.00)
Relationship between lesion and vessels	vessel passing through	0.87(20/23; 0.67,1.00)	0.96(22/23; 0.85,1.00)	1.00(19/19; 1.00,1.00)	1.00(19/19; 1.00,1.00)	0.93(39/42; 0.81,1.00)	0.98(41/42; 0.92,1.00)
	vessel passing by	0.91(20/22; 0.69,1.00)	0.91(20/22; 0.69,1.00)	1.00(20/20; 1.00,1.00)	1.00(20/20; 1.00,1.00)	0.95(40/42; 0.84,1.00)	0.95(40/42; 0.84,1.00)
	vessel distortion or dilation	0.83(5/6; 0.50,1.00)	0.83(5/6; 0.50,1.00)	1.00(36/36; 1.00,1.00)	1.00(36/36; 1.00,1.00)	0.98(41/42; 0.92,1.00)	0.98(41/42; 0.92,1.00)
	Interlobular fissure invasion	0.79(11/14; 0.50,1.00)	0.79(11/14; 0.50,1.00)	1.00(28/28; 1.00,1.00)	1.00(28/28; 1.00,1.00)	0.93(39/42; 0.81,1.00)	0.93(39/42; 0.81,1.00)

Pleural interrelationship	Pleural adhesion/retraction	0.80(20/25; 0.61,0.95)	0.80(20/25; 0.61,0.95)	1.00(17/17; 1.00,1.00)	1.00(17/17; 1.00,1.00)	0.88(37/42; 0.74,0.98)	0.88(37/42; 0.74,0.98)
	Pleural effusion	1.00(3/3; 1.00,1.00)	1.00(3/3; 1.00,1.00)	1.00(39/39; 1.00,1.00)	1.00(39/39; 1.00,1.00)	1.00(42/42; 1.00,1.00)	1.00(42/42; 1.00,1.00)

UTEu, unenhanced spiral-UTE; UTEe, delayed post-contrast spiral-UTE; CI, confidence interval. Data are presented as proportion (n/N; 95% CI). Morphology-level analyses were restricted to lesions successfully visualized on both acquisitions within the corresponding consensus panel for the paired UTEu–UTEe comparison.

Table S2 Conditional morphology-level performance among UTE-visible lesions by experienced reader panel at 3.0T

Morphological features		Sensitivity		specificity		accuracy	
		UTEu	UTEe	UTEu	UTEe	UTEu	UTEe
Margin	smooth	0.81(26/32; 0.62,1.00)	0.81(26/32; 0.62,1.00)	0.82(32/39; 0.66,0.95)	0.85(33/39; 0.69,0.98)	0.82(58/71; 0.69,0.95)	0.83(59/71; 0.71,0.96)
	lobulation	0.96(25/26; 0.86,1.00)	0.96(25/26; 0.86,1.00)	0.96(43/45; 0.88,1.00)	0.96(43/45; 0.88,1.00)	0.96(68/71; 0.90,1.00)	0.96(68/71; 0.90,1.00)
	spiculation	0.68(13/19; 0.45,0.90)	0.74(14/19; 0.50,0.94)	1.00(52/52; 1.00,1.00)	1.00(52/52; 1.00,1.00)	0.92(65/71; 0.82,0.98)	0.93(66/71; 0.84,0.99)
	irregular	0.84(26/31; 0.69,0.97)	0.84(26/31; 0.69,0.97)	0.97(39/40; 0.93,1.00)	0.97(39/40; 0.93,1.00)	0.92(65/71; 0.85,0.98)	0.92(65/71; 0.85,0.98)
Dominant shape	round	0.88(14/16; 0.58,1.00)	0.88(14/16; 0.58,1.00)	0.98(54/55; 0.94,1.00)	0.98(54/55; 0.94,1.00)	0.96(68/71; 0.90,1.00)	0.96(68/71; 0.90,1.00)
	oval	0.81(22/27; 0.57,1.00)	0.81(22/27; 0.57,1.00)	0.91(40/44; 0.79,0.98)	0.91(40/44; 0.79,0.98)	0.87(62/71; 0.77,0.96)	0.87(62/71; 0.77,0.96)
Cavitation	Central hypo-intensity of lesion	0.67(16/24; 0.38,0.89)	0.75(18/24; 0.48,0.96)	0.98(46/47; 0.93,1.00)	0.98(46/47; 0.93,1.00)	0.87(62/71; 0.78,0.95)	0.90(64/71; 0.82,0.97)
	Perilesional lucency	0.43(3/7; 0.00,0.83)	0.57(4/7; 0.20,1.00)	1.00(64/64; 1.00,1.00)	0.97(62/64; 0.91,1.00)	0.94(67/71; 0.88,0.99)	0.93(66/71; 0.86,0.98)

	Air-bronchogram	0.92(22/24; 0.83,1.00)	0.92(22/24; 0.83,1.00)	0.98(46/47; 0.93,1.00)	1.00(47/47; 1.00,1.00)	0.96(68/71; 0.91,1.00)	0.97(69/71; 0.93,1.00)
Relationship between lesion and vessels	vessel passing through	0.62(29/47; 0.40,0.85)	0.68(32/47; 0.45,0.92)	0.96(23/24; 0.88,1.00)	1.00(24/24; 1.00,1.00)	0.73(52/71; 0.57,0.89)	0.79(56/71; 0.61,0.95)
	vessel passing by	0.63(17/27; 0.41,0.86)	0.63(17/27; 0.41,0.86)	1.00(44/44; 1.00,1.00)	1.00(44/44; 1.00,1.00)	0.86(61/71; 0.77,0.96)	0.86(61/71; 0.77,0.96)
	vessel distortion or dilation	0.38(3/8; 0.00,0.75)	0.62(5/8; 0.29,1.00)	1.00(63/63; 1.00,1.00)	1.00(63/63; 1.00,1.00)	0.93(66/71; 0.87,0.98)	0.96(68/71; 0.92,1.00)
Pleural interrelationship	Interlobular fissure invasion	0.53(8/15; 0.22,0.75)	0.60(9/15; 0.33,0.83)	1.00(56/56; 1.00,1.00)	1.00(56/56; 1.00,1.00)	0.90(64/71; 0.81,0.97)	0.92(65/71; 0.84,0.98)
	Pleural adhesion/retraction	0.78(35/45; 0.62,0.93)	0.80(36/45; 0.65,0.96)	0.96(25/26; 0.86,1.00)	0.96(25/26; 0.86,1.00)	0.85(60/71; 0.74,0.94)	0.86(61/71; 0.75,0.96)
	Pleural effusion	1.00(5/5; 1.00,1.00)	1.00(5/5; 1.00,1.00)	1.00(66/66; 1.00,1.00)	1.00(66/66; 1.00,1.00)	1.00(71/71; 1.00,1.00)	1.00(71/71; 1.00,1.00)

UTEu, unenhanced spiral-UTE; UTEe, delayed post-contrast spiral-UTE; CI, confidence interval. Data are presented as proportion (n/N; 95% CI). Morphology-level analyses were restricted to lesions successfully visualized on both acquisitions within the corresponding consensus panel for the paired UTEu–UTEe comparison.

Table S3 Inter-panel agreement for nodule morphologic depiction for spiral-UTE at 1.5T and 3.0T

Morphologic characteristics		1.5T				3.0T			
		UTEu		UTEe		UTEu		UTEe	
		k	95% CI	k	95% CI	k	95% CI	k	95% CI
Margin	smooth	0.78	0.60 ~ 0.96	0.75	0.55 ~ 0.95	0.79	0.65 ~ 0.92	0.76	0.61 ~ 0.90
	lobulation	0.70	0.52 ~ 0.89	0.74	0.56 ~ 0.91	0.78	0.63 ~ 0.92	0.78	0.63 ~ 0.92

	spiculation	1.00	1.00 ~ 1.00	1.00	1.00 ~ 1.00	0.95	0.87 ~ 1.00	0.96	0.87 ~ 1.00
	irregular	0.75	0.58 ~ 0.92	0.72	0.55 ~ 0.89	0.97	0.93 ~ 1.00	0.97	0.93 ~ 1.00
Dominant shape	round	0.74	0.47 ~ 1.00	0.64	0.28 ~ 1.00	0.72	0.51 ~ 0.93	0.72	0.51 ~ 0.93
	oval	0.79	0.63 ~ 0.95	0.73	0.56 ~ 0.90	0.89	0.78 ~ 1.00	0.84	0.71 ~ 0.96
Cavitation	Central hypo-intensity of lesion	0.72	0.49 ~ 0.95	0.78	0.58 ~ 0.98	0.89	0.76 ~ 1.00	0.86	0.73 ~ 0.99
	Perilesional lucency	-*	-*	-*	-*	0.85	0.57 ~ 1.00	1.00	1.00 ~ 1.00
	Air-bronchogram	0.95	0.84 ~ 1.00	0.95	0.84 ~ 1.00	0.91	0.81 ~ 1.00	0.97	0.91 ~ 1.00
Relationship between lesion and vessels	vessel passing through	0.93	0.83 ~ 1.00	0.85	0.72 ~ 0.99	0.83	0.70 ~ 0.95	0.93	0.85 ~ 1.00
	vessel passing by	0.67	0.47 ~ 0.87	0.72	0.53 ~ 0.91	0.71	0.47 ~ 0.95	0.80	0.63 ~ 0.97
	vessel distortion or dilation	0.73	0.39 ~ 1.00	0.73	0.39 ~ 1.00	0.80	0.40 ~ 1.00	0.74	0.40 ~ 1.00
Pleural interrelationship	Interlobular fissure invasion	0.94	0.83 ~ 1.00	0.94	0.83 ~ 1.00	0.85	0.64 ~ 1.00	0.79	0.55 ~ 1.00
	Pleural adhesion/pleural retraction	0.85	0.71 ~ 0.99	0.89	0.76 ~ 1.00	0.87	0.76 ~ 0.97	0.85	0.74 ~ 0.95
	Pleural effusion	0.79	0.40 ~ 1.00	0.79	0.40 ~ 1.00	1.00	1.00 ~ 1.00	1.00	1.00 ~ 1.00

*UTEu, unenhanced spiral-UTE; UTEe, contrast-enhanced spiral-UTE; 95%CI, 95% confidence interval; * At 1.5T, no perilesional lucency was detected in any nodules of inexperienced panel, whereas only one lesion with perilesional lucency was found in experienced panel. This resulted in insufficient data for a 2×2 cross-tabulation analysis.*
